# Landscape fragmentation overturns classical metapopulation thinking

**DOI:** 10.1073/pnas.2303846121

**Published:** 2024-05-06

**Authors:** Yun Tao, Alan Hastings, Kevin D. Lafferty, Ilkka Hanski, Otso Ovaskainen

**Affiliations:** ^a^Department of Ecology, Evolution, and Marine Biology, University of California, Santa Barbara, CA 93117; ^b^Institute of Bioinformatics, University of Georgia, GA 30602; ^c^Department of Environmental Science and Policy, University of California, Davis, CA 95616; ^d^Santa Fe Institute, NM 87501; ^e^U.S. Geological Survey, Western Ecological Research Center, CA 93106; ^f^Marine Science Institute, University of California, Santa Barbara, CA 93117; ^g^Organismal and Evolutionary Biology Research Programme, Faculty of Biological and Environmental Sciences, University of Helsinki, Helsinki 00014, Finland; ^h^Department of Biological and Environmental Science, University of Jyväskylä, Jyväskylä FI-40014, Finland; ^i^Department of Biology, Centre for Biodiversity Dynamics, Norwegian University of Science and Technology, Trondheim N-7491, Norway

**Keywords:** metapopulation, fragmentation, population dynamics, landscape ecology

## Abstract

Habitat loss and isolation caused by landscape fragmentation represent a growing threat to global biodiversity. Existing theory suggests that the process will lead to a decline in metapopulation viability. However, since most metapopulation models are restricted to simple networks of discrete habitat patches, the effects of real landscape fragmentation, particularly in stochastic environments, are not well understood. To close this major gap in ecological theory, we developed a spatially explicit, individual-based model applicable to realistic landscape structures, bridging metapopulation ecology and landscape ecology. This model reproduced classical metapopulation dynamics under conventional model assumptions, but on fragmented landscapes, it uncovered general dynamics that are in stark contradiction to the prevailing views in the ecological and conservation literature. Notably, fragmentation can give rise to a series of dualities: a) positive and negative responses to environmental noise, b) relative slowdown and acceleration in density decline, and c) synchronization and desynchronization of local population dynamics. Furthermore, counter to common intuition, species that interact locally (“residents”) were often more resilient to fragmentation than long-ranging “migrants.” This set of findings signals a need to fundamentally reconsider our approach to ecosystem management in a noisy and fragmented world.

## Significance

Maintaining metapopulation viability on fragmented landscapes is of great interest in ecology and conservation biology. Although extensive theories have been developed, past models typically assume simple landscape structures that do not reflect realistic fragmentation. This major gap in ecological theory limits our ability to understand, anticipate, and mitigate ecological responses to habitat destructions in the coming decades. Here, we developed a metapopulation model that incorporates simple and complex landscape structures. We found that classical results from past models are not generalizable: The dynamics that emerge on more realistically fragmented landscapes often invalidate or reverse conventional metapopulation thinking. Our results thus represent a philosophical departure from current ideas for managing species persistence, biodiversity, and ecosystem resilience.

Habitat loss and isolation caused by landscape fragmentation pose a threat to global biodiversity even more pressing than long-term climate change (1). Initiatives to protect at least 30% of the Earth’s biomes by 2030 have recently been proposed to alleviate the myriad effects of rapidly changing landscape structures (2). Indeed, for the past 50 y, metapopulation theory has provided rich insights into the role of landscape structure by predicting how a set of spatially disjunct populations can persist in a balance between stochastic local extinctions and recolonizations (3). Earlier studies found that, in stochastic environments, landscape fragmentation can accelerate global (metapopulation) extinction (4, 5), reduce metapopulation abundance (6, 7), and inhibit spatial synchrony (8, 9). However, such generalizations were drawn mostly from models using simple networks of discrete habitat patches, which bear little resemblance to many natural landscape structures characterized by arbitrary configurations of variably sized, possibly contiguous habitat fragments. This form of spatial abstraction constrains the efficacy of metapopulation theory in addressing actual land use changes, especially when compared to the many spatially realistic population models that have been developed in the field of landscape ecology [refs. 10 and 11 (and references therein)]. The latter studies are not without their own limitations: Despite the inclusion of landscape complexity, they often exclude mechanisms that are essential to understanding metapopulation dynamics, such as density-dependent population regulation and environmental stochasticity. Here, by emphasizing the importance of landscape elements in the context of metapopulation processes, we seek to broaden metapopulation theory, extending it in the direction of landscape ecology in an attempt to encompass “real-world” fragmentation scenarios.

Metapopulation theory aims to answer fundamental questions about landscape fragmentation. For example, a) How do individual-level behaviors influence metapopulation-level responses to fragmentation? b) How do the effects of fragmentation and environmental variability (stochasticity) interact? c) Which species most need protection as fragmentation intensifies? Past efforts to resolve these questions have been hindered by the need to simplify models. In particular, reaction–diffusion models (12) tend to overlook landscape structure and apply environmental averages. Stochastic patch occupancy models (7, 13, 14) usually neglect individual-level behaviors and describe only extreme forms of environmental stochasticity (i.e., it is either locally randomized or globally synchronized). These models have long shaped classical ecological thinking, which suggests prima facie that spatial localization of ecological interactions generally have adverse effects on metapopulation viability across various environments (15–17). How closely this conclusion aligns with metapopulation dynamics on realistic landscapes remains unclear (18, 19).

Individual-based simulation models (20–22) have been the de facto approach for capturing a larger set of ecological complexities. General insights are nevertheless difficult to attain due to the trade-offs between model complexity and generality (7, 23), a problem that has contributed to a disconnect between model predictions and conservation planning. Here, we present an individual-based model that is applicable to arbitrarily structured landscapes. It achieves both model complexity and generality using optimized simulations and unique data visualizations, allowing metapopulation dynamics to be explored over a much wider range of assumptions and parameter space than previous models. Furthermore, by simulating individual-level ecological interactions over random distances, we free the spatial domains in which local dynamics occur from patch identity (24, 25). Our model is therefore able to account for environmental variation even within a continuous piece of habitat, bridging the gap between metapopulation ecology and landscape ecology.

Metapopulation dynamics have been expressed in multiple ways. Classical metapopulation models tend to solve for compound, patch-level quantities, e.g., proportion of patch (site) occupancy (14, 17), net replacement number (26, 27) and metapopulation capacity (28). These outputs yield coarse-grained, dynamically simplified interpretations that are often contingent on rigid assumptions, such as local carrying capacities always being reached between successive dispersal events (19, 29). It has thus been argued that, for conservation planning on fragmented landscapes, the prevailing models hold more heuristic than practical value (19). In an effort to advance classical metapopulation theory beyond such a limitation, we evaluate metapopulation dynamics in terms of a) mean-time to global extinction, b) global population density, and c) spatial correlations in local population dynamics, all measured on the basis of individuals rather than patches. These measurements are more testable, carry fewer assumptions, and together form an integrative, fine-grained analysis of metapopulation dynamics.

**Fig. 1.**
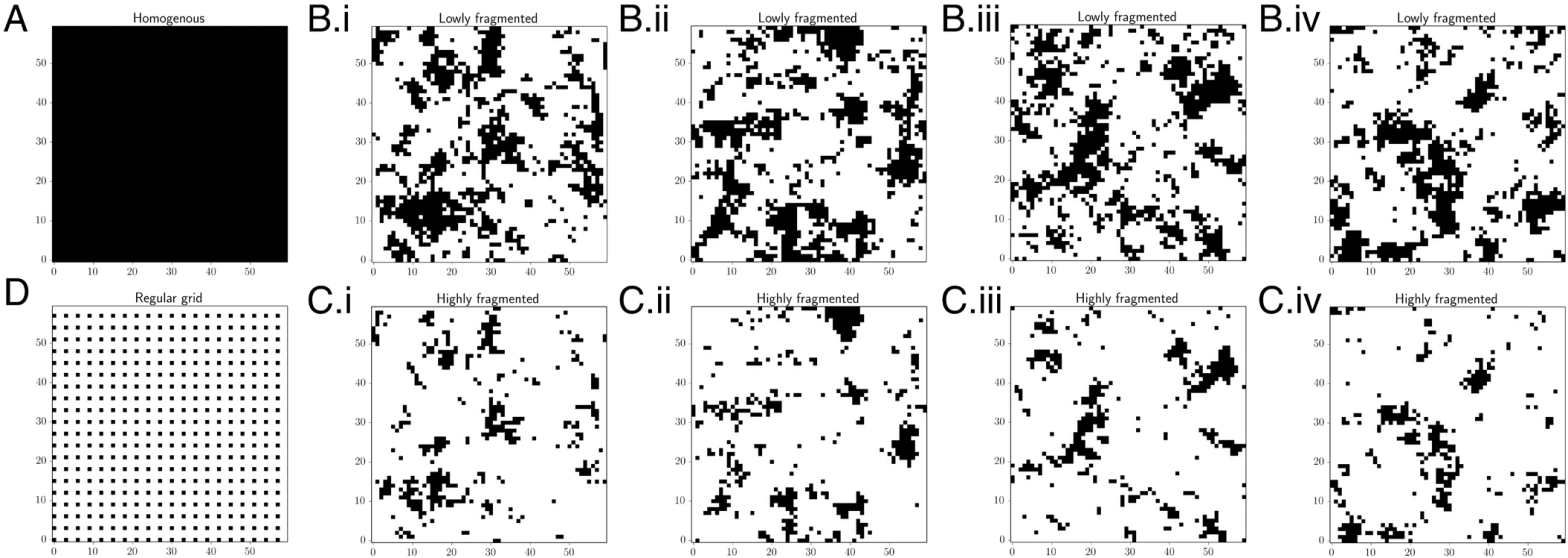
Four types of landscape structure generated on a 60 × 60 lattice, with the proportional habitat covers listed in parentheses: (*A*) homogenous (1), (*B*, *i*–*iv*) lowly fragmented (0.28, 0.30, 0.28, 0.25), (*C*, *i*–*iv*) highly fragmented (0.11, 0.13, 0.12, 0.10), and (*D*) regular grid (0.11). The darkened areas represent habitat space. In *D*, each patch has unit length and equal nearest-neighbor distances.

We examine a series of two-dimensional landscapes with structures that vary from uniform and simple to disordered and complex (Fig. 1). Throughout this paper, unless otherwise stated, “fragmented” denotes landscapes composed of a random, irregular assembly of habitat parcels. It must also be noted that, within the context of our model, “fragmentation” is interpreted as an ecological *process* that incrementally reduces *both* habitat area and connectivity, such that habitat loss and isolation are positively correlated on fragmented landscapes. This interpretation enables a direct examination of how progressive landscape degradation is likely to influence metapopulation dynamics over time, providing information valuable to improving ecological forecasts (30). To avoid conceptual ambiguity, we introduce the term “fragmentation per gradus” to distinguish our model’s definition of “fragmentation” from “fragmentation per se”, a widely discussed concept in the landscape ecology literature that refers exclusively to the mechanistic function of habitat configuration, independent of the effects of total habitat area (11).

Our model is sufficiently general to reproduce classical metapopulation dynamics as a special case (see details and results in the *SI Appendix*).

## Results

### Metapopulation Persistence.

**Fig. 2.**
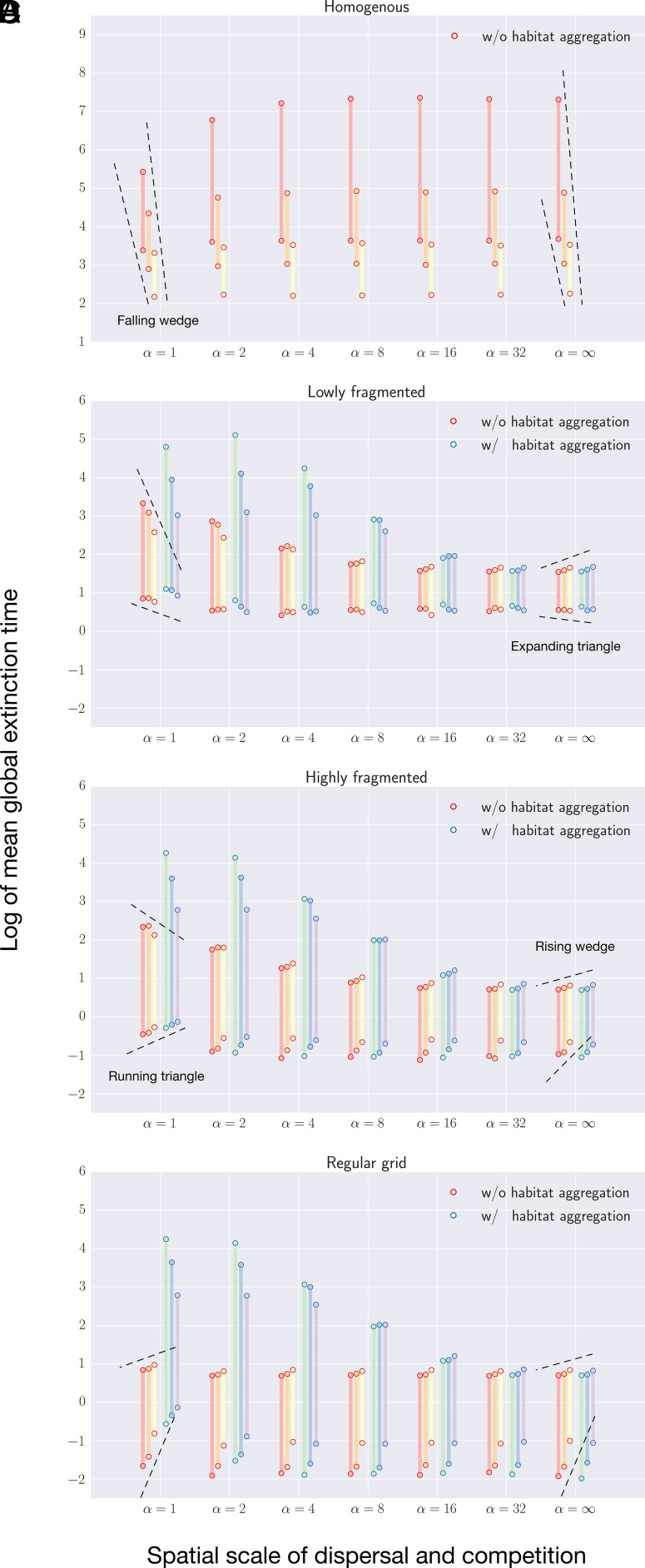
Mean times to global extinction of conspecifics on four landscapes: (*A*) homogenous, (*B*) lowly fragmented, as pictured in Fig. 1 *B*, *i*, (*C*) highly fragmented, as pictured in Fig. 1 *C*, *i*, and (*D*) regular grid, plotted on a logarithmic scale. The model domains are the full-sized landscapes in Fig. 1 (top circles) and random samples of 6 × 6 lattices (bottom circles). Conspecifics vary in their spatial scale of dispersal and competition α, ranging from 1 to ∞. The rightmost column (α=∞) thus describes the effects of total habitat area on metapopulation persistence, independent of habitat arrangement. Environmental stochasticity was globally synchronized and modeled across three values of variance $\sigma_{r}^{2}$ distinguished by line colors: 0.5 (red, green), 1 (orange, blue), and 2 (yellow, purple). In *B*–*D*, the results with and without habitat aggregation (i.e., collecting the habitat fragments into a square patch versus keeping the original habitat arrangements) are represented by blue and red circle outlines, respectively. 3,000 simulation iterations were run per system setting, each initialized with the same density of spatially randomized individuals per unit area (1,000/3,600) and continued until the metapopulation reaches global extinction or the terminal time of $5\times 10^{5}$ generations. Mean-fecundity rate $\mu_{0}=ln\left(1.1\right)$; competition strength b=0.2. Results for landscapes depicted in Fig. 1 *B* and *C*, *ii*–*iv* can be found in *SI Appendix*, Fig. S5*A*.

On homogenous landscapes, species that disperse and compete locally (over short distances), henceforth termed “residents,” went extinct the fastest, likely because of overcrowding or underexploitation (Fig. 2*A* and *SI Appendix*, Fig. S2*A*). Increased environmental noise (variance of stochasticity) accelerates metapopulation extinction, as depicted by the trend lines forming a “falling wedge” pattern (Fig. 2*A*).

Fragmented landscapes, by contrast, promote faster extinction of “migrants,” our shorthand for species that disperse and compete over long distances. Interestingly, here, environmental noise can have diverging effects on persistence time depending on the area of the landscape (cf. Fig. 2 *A*–*C*). For instance, on lowly fragmented landscapes, increasing environmental noise is detrimental to the survival of migrants on small landscapes, but the same process delays extinction on large landscapes, a duality captured by the “expanding triangle” pattern (Fig. 2*B* and *SI Appendix*, Fig. S2*B*). The latter feature conflicts with the general assumption that a metapopulation would have a lower risk of going extinct in a constant, rather than fluctuating, environment (31). On highly fragmented landscapes, alternative metapopulation responses emerge (Fig. 2*C* and *SI Appendix*, Fig. S2*C*). Resident persistence follows a “running triangle,” an inverse pattern indicating noise-induced delay (acceleration) of metapopulation extinction on small (large) landscapes (Fig. 2*C*). For migrants, the “expanding triangles” are replaced by “rising wedges,” reflecting noise-induced extinction delay irrespective of landscape area (Fig. 2*C*).

On a regular grid landscape, residents and migrants are nearly equally persistent (Fig. 2*D* and *SI Appendix*, Fig. S2*D*). The effect of environmental noise is opposite to that on homogenous landscapes (cf. Fig. 2 *A* and *D*). Moreover, a comparison to the outcomes on the highly fragmented landscape of similar total habitat areas (cf. Fig. 2 *C* and *D*) shows how assuming simple patch networks can greatly underestimate resident persistence on “real” landscapes.

From a conservation planning perspective, habitat aggregation (the opposite of fragmentation per se) can markedly prolong metapopulation persistence, partially compensating for the adverse effect of fragmentation. This strategy is most effective for conserving residents inhabiting large landscapes (Fig. 2 and *SI Appendix*, Fig. S2). The gain in persistence time is usually smaller in noisier environments (Fig. 2 *B*–*D*).

### Metapopulation Abundance.

**Fig. 3.**
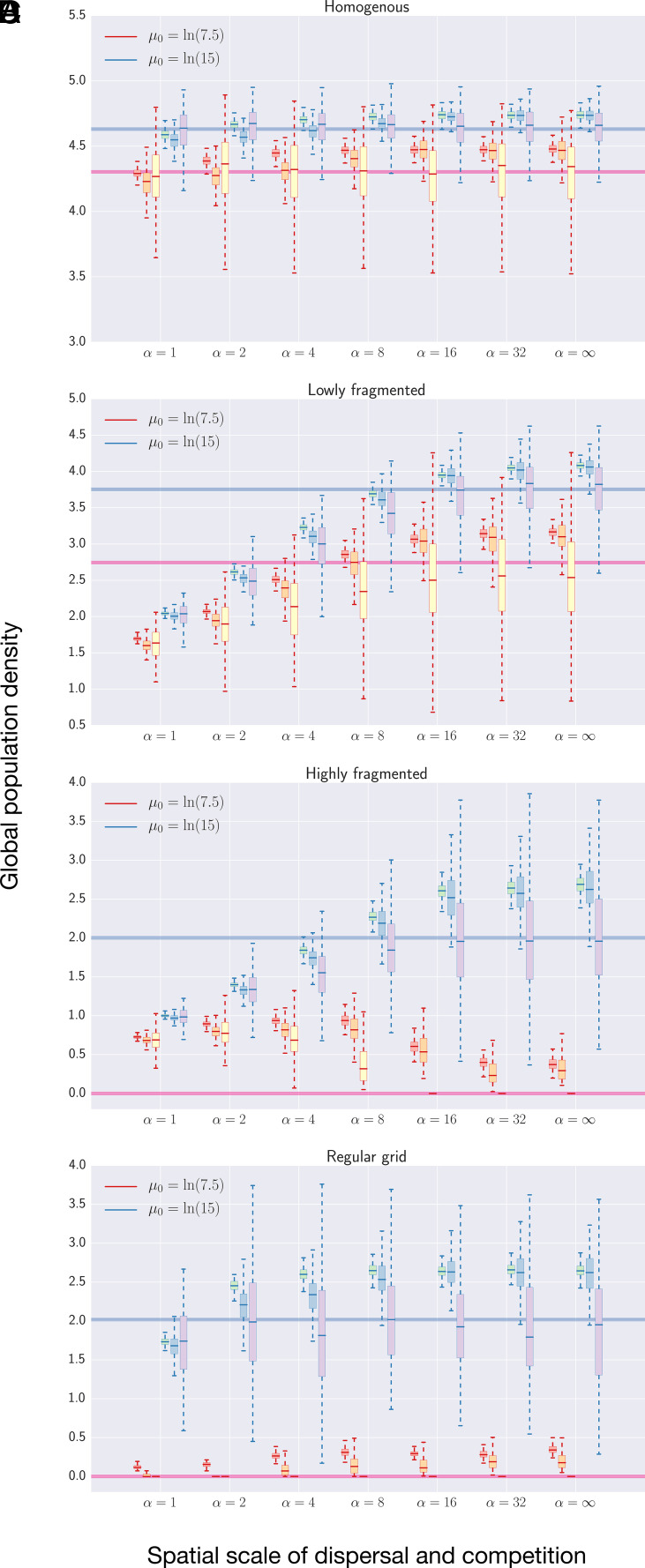
Global population densities of conspecifics on four landscapes: (*A*) homogenous, (*B*) lowly fragmented, as pictured in Fig. 1 *B*, *i*, (*C*) highly fragmented, as pictured in Fig. 1 *C*, *i*, and (*D*) regular grid. Conspecifics vary in their spatial scales of dispersal and competition α, ranging from 1 to ∞, and mean fecundity rates $\mu_{0}$, marked by the colors of the box outlines (red and blue). Regional stochasticity was modeled across three values of spatial scale $\alpha_{r}$, indicated by the filled colors: 1.5 (red, green), 6 (orange, blue), and ∞ (yellow, purple). Five iterations were run per parameter set, each initialized with 5,000 spatially randomized individuals and tracked for 250 generations, with only the last 50 generations shown. The horizontal lines, colored to match $\mu_{0}$, depict time-variant mean-field model predictions averaged across five simulation iterations and the last 50 of 250 generations. Variance of regional stochasticity $\sigma_{r}^{2}=0.5$; competition strength b=0.2. Results for landscapes depicted in Fig. 1 *B* and *C*, *ii*–*iv* can be found in *SI Appendix*, Fig. S5*B*.

As expected, fragmentation reduces the abundance of all species through habitat loss. However, interspecific differences emerge over time in ways that are uniquely dependent on the level of fragmentation (Fig. 3 *A*–*C* and *SI Appendix*, Fig. S3 *A*–*C*). For instance, low-level fragmentation caused greater declines in resident abundances (Fig. 3*B*), whereas high-level fragmentation can impact migrants more than residents of equal reproductive output, driving the former to global extinction especially in stochastic environments where the habitat qualities are broadly correlated (Fig. 3*C*). Such time-varying hierarchies in metapopulation performance suggest that some life-history traits that were initially more fragmentation-resistant (e.g., long-distance dispersal at the expense of propagule number) could eventually become maladaptive (32). These results could not be accurately captured by conventional models that assume mass-action mixing, e.g., stochastic Beverton–Holt predictions (Fig. 3 *B* and *C* and *SI Appendix*, Fig. S3 *B* and *C*).

On a regular grid landscape, interspecific differences were minor (Fig. 3*D* and *SI Appendix*, Fig. S3*D*). Instead, we found more significant differences when compared to the highly fragmented landscape (cf. Fig. 3 *C* and *D*). The disparities between these two landscape structures reinforce the importance of describing realistic habitat arrangements for ecological predictions and inferences.

### Metapopulation Synchrony.

**Fig. 4.**
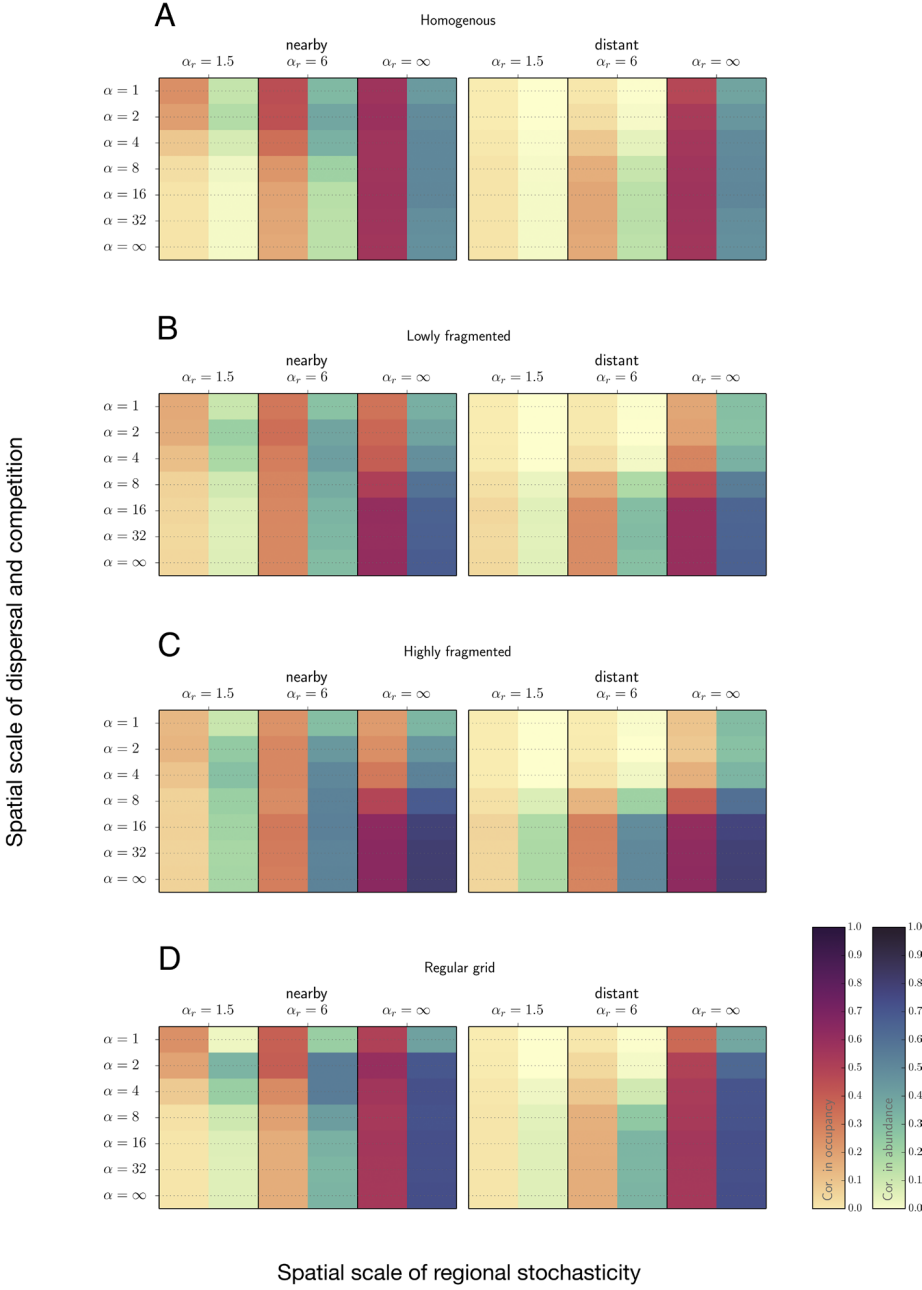
Interlaced correlograms showing spatial synchrony on four landscapes: (*A*) homogenous, (*B*) lowly fragmented, as pictured in Fig. 1 *B*, *i*, (*C*) highly fragmented, as pictured in Fig. 1 *C*, *i*, and (*D*) regular grid. Synchrony was measured by the pairwise correlations in local patch occupancy and local abundance at the final generation, illustrated using distinct color gradients. Sampling sites are identical square grids of length m=2. The left panels show the levels of synchrony between nearby populations (separated by an intersite distance $l_{\mathrm{ij}}\approx 2$); the right panels show those between distant populations ($l_{\mathrm{ij}}\approx 20$). Mean fecundity rates $\mu_{0}$ were adjusted to maintain a near-constant mean global population size N (1,000 ± 200) across all combinations of landscape structure, spatial scale of regional stochasticity $\alpha_{r}$, and spatial scale of dispersal and competition α. 5,000 simulation iterations were run per parameter set, each initialized with 5,000 spatially randomized individuals and tracked for 50 generations. Variance of regional stochasticity $\sigma_{r}^{2}=0.5$; competition strength b=0.2. Results for landscapes depicted in Figs. 1 *B* and *C*, *ii*–*iv* can be found in *SI Appendix*, Fig. S5*C*.

Fragmentation can have both synchronizing and desynchronizing effects. As fragmentation progresses, the strength of spatial synchrony varies across species in a manner we term synchronic flow, a remark on its likeness to mass redistribution by means of convective transport (cf. Fig. 4 *A*–*C*). Typically, this process increases synchronies between migrant populations while reducing those between resident populations (cf. Fig. 4 *A*–*C* and *SI Appendix*, Fig. S4 *A*–*C*). These interspecific divergences become more pronounced when local environmental conditions are correlated over large distances (Fig. 4*C* and *SI Appendix*, Fig. S4*C*).

The overall pattern of synchrony on the homogenous landscape is commensurable to that on the regular grid landscape despite a ninefold difference in habitat availability (Fig. 4 *A* and *D* and *SI Appendix*, Fig. S4 *A* and *D*). In contrast, there is a qualitative difference between the regular grid and the highly fragmented landscape (cf. Fig. 4 *C* and *D*). We can accordingly conclude that synchronic flow is unlikely a model artifact due to variation in habitat availability (or population density per habitat unit) but is rather a general phenomenon driven by fragmentation.

The preferred method for measuring synchrony may vary over the course of the fragmentation timeline. Synchrony becomes more detectable through correlations in abundance than in occupancy following habitat loss. Meanwhile, the area-effect of sampling sites diminishes (*SI Appendix*, Fig. S4).

## Discussion

An individual-based model that incorporates arbitrary landscape structures makes it possible to explore the effects of landscape fragmentation (i.e., fragmentation per gradus) on metapopulation persistence, abundance, and synchrony under various combinations of species-specific and environment-specific parameters. Our model can reproduce classical metapopulation dynamics on a simple landscape. Under fragmentation, the general predictions starkly contradict classical ecological thinking, suggesting that applications of conventional metapopulation models to “real” landscapes may lead to spurious conclusions regarding metapopulation resilience in the face of landscape transformation. Given that the fundamental mechanisms of our model are general, its findings can be applied to a wide range of taxa where dispersal and offspring competition are intrinsic features of the system.

On fragmented landscapes, some metapopulations persisted longer in noisier environments. This phenomenon of noise-induced persistence, which is evocative of the “inflationary” effect described by Roy et al. (33), is particularly evident in systems characterized by numerous “ditch sites”—habitat-poor destination hubs where individuals are removed en masse from the global population pool. The prevalence of ditch sites is influenced by two critical factors related to landscape connectivity: 1) the spatial distribution of dispersers in the “airspace,” and 2) the probability of those dispersers landing in suitable habitats. We contrast ditch sites with “dock sites”: destination hubs that are habitat-rich. These two terms resonate with the concept of source–sink habitats and provide a fresh interpretative framework. For example, on highly fragmented landscapes, environmental noise enhances metapopulation persistence of long-ranging species because the system’s facilitation of mass dispersals into inhospitable regions favors the formation of ditch sites over dock sites.

As landscape area decreases, noise-induced persistence can turn into noise-induced extinction, or vice versa. The reason for this becomes apparent when we consider that imposing periodic boundary conditions on a subsampled region may significantly alter the “dock-ditch” ratio on the original landscape, either by a) “docking” more long-distance dispersers on their parental habitats or, if the landscape is highly fragmented, b) “ditching” more local dispersers near landscape edges. This area dependence illustrates the risk of extrapolating results from small, fragmented landscapes with wrap-around boundaries, and underscores the importance of scale-appropriate models and experimental designs.

Fragmentation can drive nonlinear changes in relative species abundance over time. In an early stage of fragmentation, resident species may see a more rapid decline in metapopulation abundance compared to migrant species, partly owing to differences in the strength of the rescue effect. However, in a later stage of fragmentation, the number of residents might surpass that of migrants, as migrants begin to suffer greater losses in the matrix. It follows that there exists a fragmentation threshold beyond which it becomes more advantageous to abstain from wasteful dispersal than to increase recruitment through immigration. This seesaw-like pattern in relative species abundance is not well-captured by mean-field approximations, lending support to Hiebler’s argument (20) that the mean-field approach often used to derive metapopulation theory has limited applicability on fragmented landscapes.

In a similar manner, landscape structure could affect species richness and community composition. Assuming that individual behaviors are nonplastic, reproduction rate is low, and interspecific interactions are negligible, fragmentation can transform species-rich communities dominated by migrants into species-poor communities dominated by residents. In addition, the combination of fragmentation and the loss of microclimates (i.e., an increase in the spatial scale of regional stochasticity) was found to drastically heighten the risk of biodiversity collapse. These metacommunity-level interpretations broadly align with current empirical research (34) and highlight the need to include microclimate maintenance as a part of conservation effort.

Geographically disjunct populations can be brought into synchrony by either dispersal or regional stochasticity through the Moran effect (24). When these mechanisms are acting together, as appears to be the case in most natural systems, disentangling their superimposed effects from the resulting pattern in the synchrony-distance relationship is non-trivial (24, 35). Also hard to ascertain is how synchrony can be further shaped by fragmentation, a topic that has received only modest attention in theory and empirical research (but see ref. 36). We showed that the shape of the synchrony-distance relationship on all types of landscapes was governed by dispersal distance in a way consistently different than it was by the spatial scale of regional stochasticity. This suggests that functional forms could be derived and fitted to landscape-specific synchrony data to help discern the contribution of each mechanism. On fragmented landscapes, statistical signatures of their relative influence might be identified through cross-species comparisons, e.g., a more positive correlation between synchrony and dispersal distance reflects a stronger Moran effect.

It is widely held that fragmentation, by increasing the distances between habitat parcels and thus the degree of habitat isolation, reduces spatial synchrony among extant populations. We tested this assumption and found that the relationship is heavily dependent on species behavior: fragmentation can indeed lead to desynchronization in resident species, but it can also drive synchrony in migrant species. We explain this phenomenon (synchronic flow) by proposing the concept of a “democratization effect,” which describes how, in the presence of multiple units of source habitats, each unit has a diluted impact on the local population dynamics at any given location. For residents, the source habitats are closely clustered and will remain mostly intact despite fragmentation. This preserves the negative effect of fragmentation on spatial synchrony. In contrast, for migrants, more source habitats are located far away; as fragmentation intensifies, accompanied by greater habitat loss, many of these distant sources are removed. Consequently, all extant source populations assume a more influential role, representing a reversal of the democratization effect (antidemocratization), and contribute to a net increase in spatial synchrony.

Standard conservation management actions may have subtle constraints when they are carried out on fragmented landscapes. For instance, expanding reserve boundaries, a costly yet popular conservation measure, might not meaningfully delay metapopulation extinction when conspecific interactions span long distances. Furthermore, habitat aggregation, a standard conservation strategy that is thought to promote metapopulation persistence in general (37), seems to mainly benefit resident species and may be ineffective when implemented over small regions or in highly variable environments.

Counter to common intuition, resident species were oftentimes *more* fragmentation-resistant than migrants (refs. 38 and 39 both hinted at the same conclusion). When fragmentation becomes severe, spatial localization of ecological interactions can readily lead to longer persistence, higher abundance, and more asynchronous local dynamics at the metapopulation level. The notion that limiting the physical distance of conspecific interaction enhances metapopulation viability signifies a philosophical departure from conventional thinking on ecosystem management. In practice, it offers a distinctive and potentially more cost-effective approach to conservation, namely, insulating vulnerable populations via movement barriers, an alternative to the standard tactics of increasing landscape connectivity and recolonization rates. We expect that such an “insulate-to-protect” strategy applies not only to terrestrial ecosystems, as suggested in the present study, but can have broad utility, e.g., informing management decisions for marine ecosystems where the impact of deep-sea mining will likely cause additional fragmentation to many already fragmented seafloor habitats (40).

Whether habitat reconfiguration alone, a factor widely termed “fragmentation per se” (11), has an overall positive or adverse effect on biodiversity and other ecological responses, including metapopulation viability, is still being fiercely debated among ecologists and conservation biologists (41, 42). Our analysis of metapopulation persistence in systems with and without habitat aggregation (while landscape size and total habitat area stay fixed) helps to clarify this issue by demonstrating that networks of few large habitats support longer-term persistence than networks of many small-to-medium habitats that have the same combined area. This directly addresses the single-large-or-several-small (SLOSS) question (43) and adds to a growing body of theory supporting the notion that, when habitat is scarce (covering less than 30% of the landscape), fragmentation per se is generally detrimental to conservation-reliant species (44). However, in communities marked by strong interspecific interactions (e.g., competition, predator–prey), both positive and negative effects have been predicted (11, 44). We also recognize the point stressed by Wiens (18) that empirical landscapes are more complex than a dichotomous habitat-matrix description and may display spatial variation in matrix hostility. Breaking a habitat patch apart and scattering the fragments across a mosaic of semihospitable matrix types can hypothetically enlarge the viable area for some long-ranging species, resulting in a positive association between fragmentation per se and metapopulation persistence (11), a topic that should be explored further given its conservation implications.

Less contentious is the debate over ordered (i.e., arranged into a regular gridwork) versus disordered (i.e., irregularly fragmented) habitat arrangements. Using a network model, Grilli et al. (45) concluded that disordered assemblies of habitat are more favorable to metapopulation viability. Our model uncovered some caveats to this rule: on irregularly fragmented landscapes, a) fecund species are equally or less abundant; and b) populations of migrants are more synchronized, hence less resilient to sudden, large-scale collapse. These findings suggest that the effects of spatial assumptions in a metapopulation model can be profound yet nuanced. Future models could a) take into account non-Euclidean dispersal distances and b) model fragmentation as a dynamic socioecological process against the backdrop of a continually evolving landscape mosaic (similar studies can be found in refs. 10 and 46).

Our results lend theoretical support to the empirical claim, recently made by Martin et al. (47), that species with strong dispersal abilities are often more vulnerable to land use intensification than species with weak dispersal abilities, further challenging the widely accepted rule that limited mobility is a reliable indicator of species risk and conservation importance. In addition, we indirectly showed that when total habitat area is kept constant, landscapes with higher densities of habitat edges generally experience more rapid metapopulation extinction, consistent with cross-taxa empirical observations on landscapes with moderate-to-high levels of habitat coverage (48). Although theoretical forecasts of species responses to fragmentation are not always in agreement with empirical findings (48, 49), our study suggests that context-dependence in ecology may help explain the discrepancies, including responses that initially appear contradictory. We also predicted substantial changes in responses across different stages of fragmentation, an indication that there is novel information to be gained by tracking fragmentation effects through atypically long-term field studies.

Metapopulation models are frequently used in epidemiology to clarify the effects of spatial and social connectivity on the spread of infectious diseases and options for intervention (50). The recent COVID-19 pandemic has underlined the additional need for models to capture the reality at the landscape scale in our preparation for present and future outbreaks. It is now known that resource scarcity and culturally rooted attitudes toward public health recommendations can create an uneven patchwork of responses, that is, spatial fragmentation in logistical or social variables, that critically influences management success (51, 52). Our present model could be modified to support epidemic planning in such complex, real-world scenarios.

In closing, our model provides a framework for understanding the effects of landscape fragmentation on metapopulation dynamics. The results bring us closer to a general form of metapopulation theory. As concerns escalate over the ecological impacts of human land-use activities in the coming decades, our predictions may be used to identify unorthodox but more effective conservation actions.

## Methods

### Landscape Generation.

We contrast a homogeneous landscape composed solely of habitat (Fig. 1*A*), fragmented landscapes, in which the proportions of irregularly shaped habitat are either moderate (24 to 30%: termed “lowly (lightly) fragmented”; Fig. 1*B*) or further reduced (10 to 13%: “highly (heavily) fragmented”; Fig. 1*C*), and a regular grid that comprises equidistant, identical square patches surrounded by the matrix, with the habitat totaling 11% of the landscape (comparable to the proportions in Fig. 1*C*), a representation of the simple patch networks assumed in classical metapopulation theory (Fig. 1*D*). To minimize finite-size effects, we imposed periodic boundary conditions. The fragmented landscapes were generated by sampling Gaussian random fields ε(x) with zero mean and variance–covariance structure

$$\mathrm{cov}(\varepsilon \left(x\right),\varepsilon (x\prime ))=\exp(-d(x,x\prime )/\alpha_{e}),$$

where d(x,x′) is the distance between locations x and x′ and the parameter $\alpha_{e}$ measures the spatial scale of landscape heterogeneity. The landscapes shown in Fig. 1 *B* and *C* were constructed using $\alpha_{e}=3$; location x was classified as habitat if $\varepsilon \left(x\right)<0.5$ and 1.1, respectively.

### Individual-based Simulations.

In each nonoverlapping generation, a semelparous individual matures and produces a Poisson distributed number of propagules (with mean μ), after which the sessile adult dies instantaneously. The propagules disperse according to a bivariate Gaussian kernel with mean zero and variance–covariance matrix $\alpha^{2}I$, where α measures the spatial scale of dispersal. Propagules that disperse into the matrix die immediately and do not influence local competition. Those that disperse into habitat experience density-dependent competition with other recruits, influencing their probability of becoming established as members of the next generation. The establishment probability of propagule i is given by $e_{i}=1/\left(1+bn_{i}\right)$; b sets the strength of competition and $n_{i}$ denotes the local propagule density. We estimated $n_{i}$ by convolving the distribution of propagules with a competition kernel sharing the same functional form as the dispersal kernel; therefore, α also measures the spatial scale of competition. Hereafter, we regard each behavioral variant α as conspecifics. We also compared species with differing scales of dispersal and competition in our sensitivity analysis (*SI Appendix*, Fig. S6). The code for simulating all individual-level processes is vectorized to improve runtimes.

To model spatially correlated environmental stochasticity, that is regional stochasticity, we assumed that the fecundity parameter μ varies over space and time. Variation was assumed to be independent among generations (no temporal autocorrelation). Fecundity was spatially correlated, such that μ is multivariate log-normally distributed with mean $\mu_{0}$ and variance–covariance structure

$$\mathrm{cov}(\log(\mu (x)),\log(\mu (x\prime )))=\sigma_{r}^{2}\exp(-d(x,x\prime )/\alpha_{r}).$$

We randomized the spatial distribution of habitat quality over time by resampling a Gaussian random field $\mu \left(x\right)$ at the start of each generation. $\alpha_{r}$ measures the spatial scale of regional stochasticity and $\sigma_{r}^{2}$ its variance (or magnitude), which we refer to as environmental noise. We note that increasing either parameter could reflect the consequence of climate change (36).

### An Integrative Analysis of Metapopulation Dynamics.

In our effort to extend metapopulation theory beyond classical dynamics, we first investigate how the classical relationships between metapopulation persistence time (i.e., mean-time to global extinction) and model parameters could be altered on fragmented landscapes. In this analysis, we sample square subregions of a given area from each landscape in Fig. 1, reset the periodic boundary condition, and simulate metapopulation dynamics until no individual remains or the generation limit is reached. This part of our analysis assumes globally synchronized environmental stochasticity $\left(\alpha_{r}=\infty \right)$, which we describe by randomizing a global value of $\mu_{0}$ per generation. In the case of mass-action mixing (α=∞), propagules disperse uniformly, and offspring competitions are influenced by global rather than local density. We also test the effects of habitat aggregation and, by extension, fragmentation per se by collecting the habitat fragments in each previously sampled subregion into a square patch and rerunning the simulations.

In our second analysis, we explore how fragmentation mediates metapopulation abundance (i.e., global population density) under different values of α and $\alpha_{r}$, with $\sigma_{r}^{2}$ held constant. Here, each simulation continues for a fixed number of generations long enough for metapopulation abundance to reach quasistationarity. Two values of $\mu_{0}$ are used to represent a contrast in reproductive output: On the habitat-poor landscapes (Fig. 1 *C* and *D*), one gives rise to global persistence and the other to global extinction. We then compare our abundance predictions to results from the stochastic mean-field (Beverton–Holt) model: $N_{t+1}={N_{t}\mu }_{t}h/\left(1+b{N_{t}\mu }_{t}h\right),$ where $N_{t}$ denotes the global population density in generation t, h is the proportion of landscape that is habitat, and $\mu_{t}$ follows the log-normal distribution with mean $\mu_{0}$ and variance $\sigma_{r}^{2}$.

In our final analysis, we estimate metapopulation synchrony in the forms of spatial correlations in local occupancy and local abundance, conditional on persistence. Each landscape is divided into a grid of m×m squares, i.e., sampling sites; neighboring sites are separated by one lattice spacing. To measure local occupancy, we determine the fraction of habitat cells that are occupied on sampling sites containing habitat. To measure local abundance, we calculate the number of occupants divided by the number of habitat cells on nonempty sites. Since any inherent spatial synchrony in the system can be confounded if, at the time of measurement, a vast majority of sampling sites is either vacant or fully occupied, we adjust $\mu_{0}$ to keep mean global population size N always within a standard, ecologically informative range. α and $\alpha_{r}$ are again varied independently.

## Supplementary Material

Appendix 01 (PDF)

## Data Availability

Our landscape dataset and simulation scripts are available in the following public repositories: https://doi.org/doi:10.5061/dryad.31zcrjdtk (53) and https://doi.org/10.5281/zenodo.10810604 (54).
