# Supplementary material for: Landscape fragmentation overturns classical metapopulation thinking
Source: Proc Natl Acad Sci U S A. 2024 May 6;121(20):e2303846121. doi: 10.1073/pnas.2303846121 (PMC11098110; doi:10.1073/pnas.2303846121)
Supplement: Supplementary file 1 — Appendix 01 (PDF) [file pnas.2303846121.sapp.pdf]

Online Appendix

We expected that with an appropriate parameterization and landscape structure, the individual-based model will generate classical metapopulation dynamics as defined by the four conditions enumerated by Hanski et al. (1): 1) discrete habitat patches support local breeding populations; 2) the local populations are prone to extinction; 3) extinctions are balanced by recolonizations due to migration from extant populations, and recolonization probability increases with increasing connectivity to potential source populations; and 4) dispersal is so infrequent that local population dynamics (local growth rates) in the extant populations are essentially independent of the states of the other populations, leading to largely asynchronous local dynamics. We hypothesized that this would require the spatial scales of ecological interactions (i.e., dispersal and competition) α and regional stochasticity α_r to be large in relation to the size of a habitat grid cell (i.e., patch), but small in comparison with inter-patch distances, and that regional stochasticity is strong enough (in relation to population density per habitat unit) to cause frequent local extinctions.

We ran the model under a suitable parameter regime for the regular grid landscape in Fig 1d and assumed that both α and α_r are greater than the length of each patch ℓ_1 and less than the shortest distance of patch separation ℓ_2. The four conditions for classical metapopulation dynamics were evaluated separately under an identical set of parameters. To test the first condition, we compiled the simulated dispersal outcomes and computed the proportions of all propagules that landed in i) their local (i.e., parental) patches, ii) nearest-neighboring patches at a distance of ℓ_2, and iii) more remote patches, as shares of total propagules that were dispersed into habitat space. For the second condition, we measured the durations of all patch occupations between local extinction events, marking a patch as occupied if at least one individual was present. We evaluated the third condition by first collecting data of all cases where a patch i is empty at time t, calculating its level of connectivity to potential source populations in other patches j, then tracking the state of its occupancy at time $t+1$. Here, connectivity is defined as $S_i(t) = \sum_{j \neq i} \exp(-d_{ij}/\alpha) o_j(t)$, where d_{ij} is the inter-patch distance from i to j, and $o_j(t) = 1$ if patch j was occupied at time t and 0 otherwise. To test the final condition, we repeated the synchrony analysis described in our Methods but defining each patch as a sampling site.

Our model, when applied to the regular grid landscape, numerically verifies the prediction that the system should behave as a classical metapopulation according to its four conditions when the spatial scales of ecological processes, α and α_r, lie between ℓ_1 and ℓ_2 (Fig S1). Our results demonstrated that more than half (58%) of all propagules dispersed into habitat space land inside their parental patches, thus indicating the capacity for discrete habitat patches to support local breeding populations (Fig S1a). Yet, within this metapopulation, patch occupation with at least one individual is typically short: the relative frequency distribution of time-to-patch-extinction shows that the majority of local populations become

extinct in less than five generations (Fig S1b). Long-term global persistence is nevertheless possible as a result of migrations from extant populations. The probability of recolonization is confirmed to increase with the degree of connectivity to potential source populations (Fig S1c). Finally, our model produced spatial synchrony patterns indicative of independent local dynamics between remote populations due to infrequent dispersal. Correlations in local patch occupancy and local abundance (i.e., population density per habitat unit) both decline with distance. Specifically, the population dynamics are coupled twice as strongly between adjacent patches as between those separated by an additional patch. When the distance between populations exceeds half the length of the landscape, their local dynamics become completely asynchronous under both measures (Fig S1d).

References

1. I. Hanski, T. Pakkala, M. Kuussaari, G. Lei, Metapopulation persistence of an endangered butterfly in a fragmented landscape. *Oikos* **72**, 21-28 (1995).

Supplementary Material

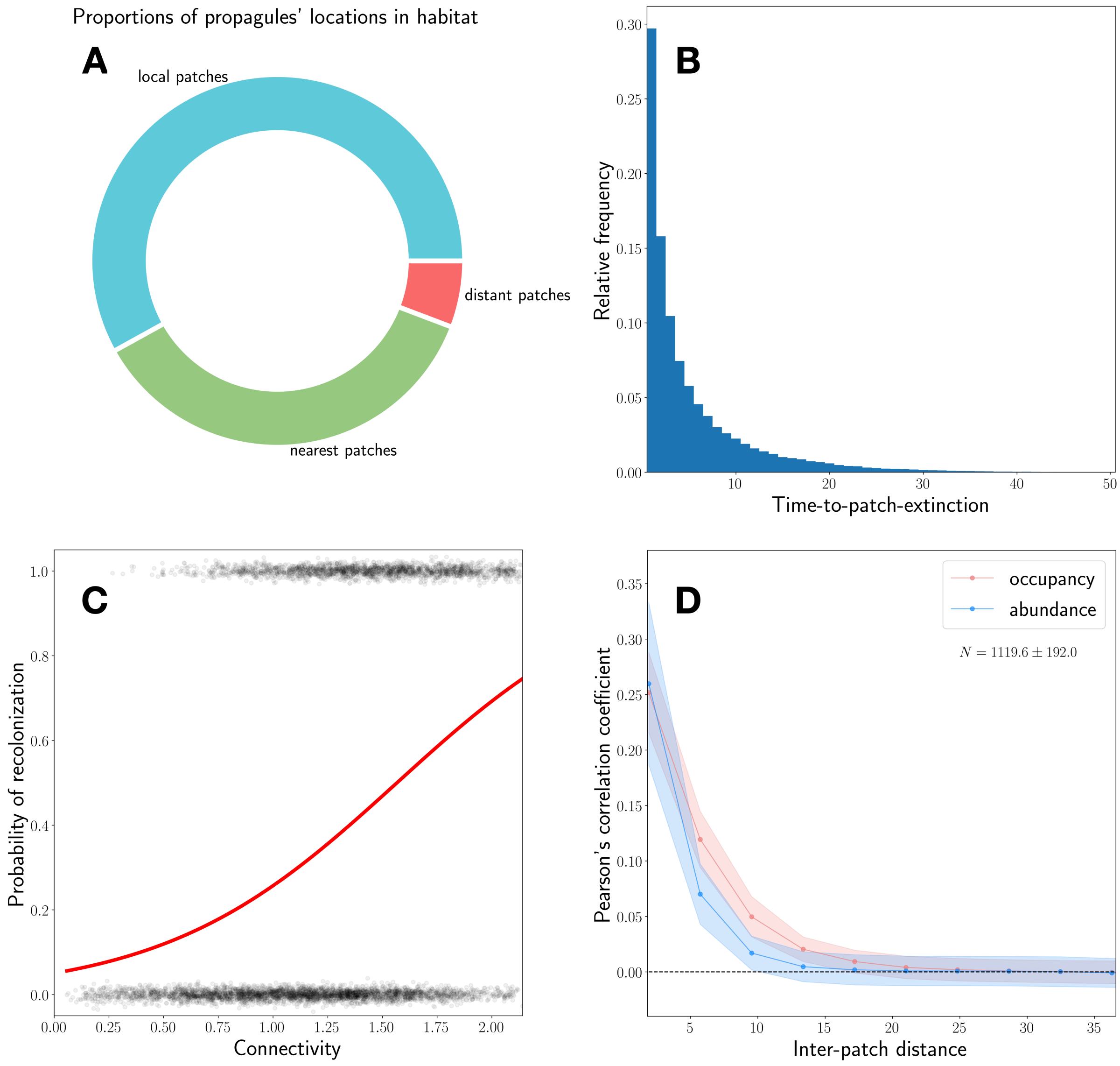

Fig S1. Reproduction of classical metapopulation dynamics as defined by four principal conditions, assessed on a regular grid landscape under regional stochasticity. a) Proportions of propagules that landed on local (i.e., parental) patches (blue), nearest patches separated by distance $\ell_2 = 2$ (green), and more distant patches (red) relative to the total number of dispersers in habitat. b) Relative frequency distribution of generational time until an occupied habitat patch becomes extinct. c) Probability of recolonizing an empty patch as a function of connectivity. Small circles indicate the simulated outcome of recolonization (0: failure; 1: success; jittered along the y-axis) given the connectivity to source populations at the previous time step and are fitted to a logistic regression (red line). d) Population synchrony as a function of distance between patches, represented by pairwise correlations in i) local patch occupancy and ii) local abundance,

i.e., population density per habitat unit, at terminal time. Circles denote correlation coefficients averaged over distance bins; the colorized boundaries their standard deviations. The broken horizontal line gives the correlational limit where dynamics are asynchronous. In all four plots, the spatial scales of dispersal and competition $\alpha = 1.5$, mean-fecundity rate $\mu_0 = \ln(8)$, competition strength $b = 0.2$, and regional stochasticity exists at spatial scale $\alpha_r = 1.5$ with variance $\sigma_r^2 = 0.5$. 1000, 100, 1, and 10000 simulation iterations are run in (a-d), respectively, each initialized with 5000 randomized individuals and continued for 50 generations.

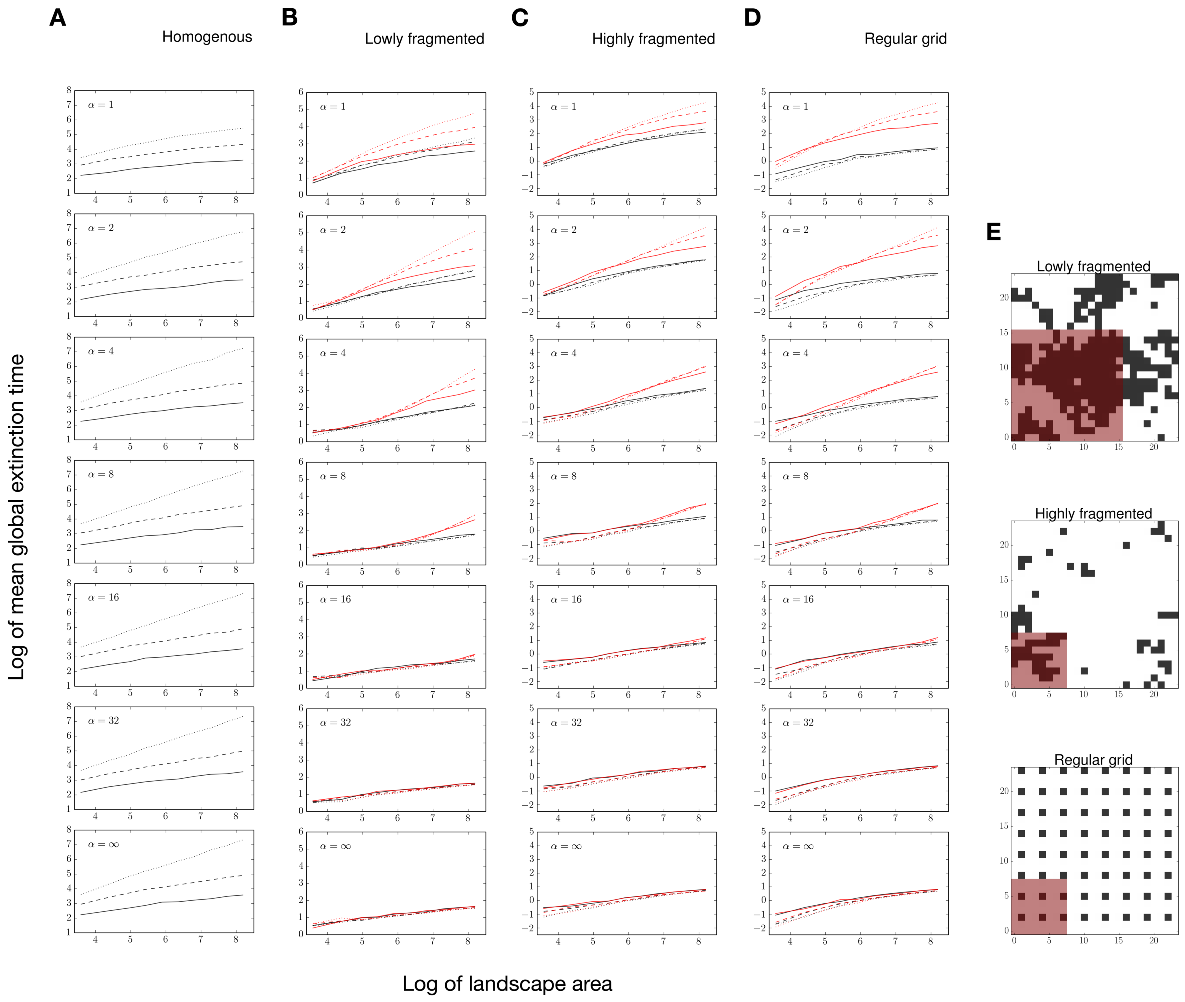

Fig S2. Mean times to global extinction of conspecifics as a function of landscape area on four landscapes: a) homogenous, b) lowly fragmented, as pictured in Fig 1b.i, c) highly fragmented, as pictured in Fig 1c.i and d) regular grid, plotted on a double logarithmic scale. Spatial scale of dispersal and competition α varied from 1 to ∞. Environmental stochasticity was globally synchronized and modeled across three values of variance σ_r^2: 0.5 (dotted), 1 (dashed), and 2 (solid). For the three non-homogenous landscapes, simulations are performed on square lattices sampled randomly from the full-sized landscapes, with lattice sizes varying from 6 x 6 to 60 x 60. The results with and without habitat aggregation are shown in red and black, respectively. 1000 simulation iterations were run per system setting, each initialized with 1000/3600 spatially randomized individuals per unit area and continued until the metapopulation reaches global extinction or the terminal time of $5\text{x}10^5$ generations. Mean-fecundity rate $\mu_0 = \ln(1.1)$; competition strength $b = 0.2$. e) Examples of habitat aggregation on 24 x 24 lattices sampled from three types of landscapes, with the aggregated square patches highlighted in red.

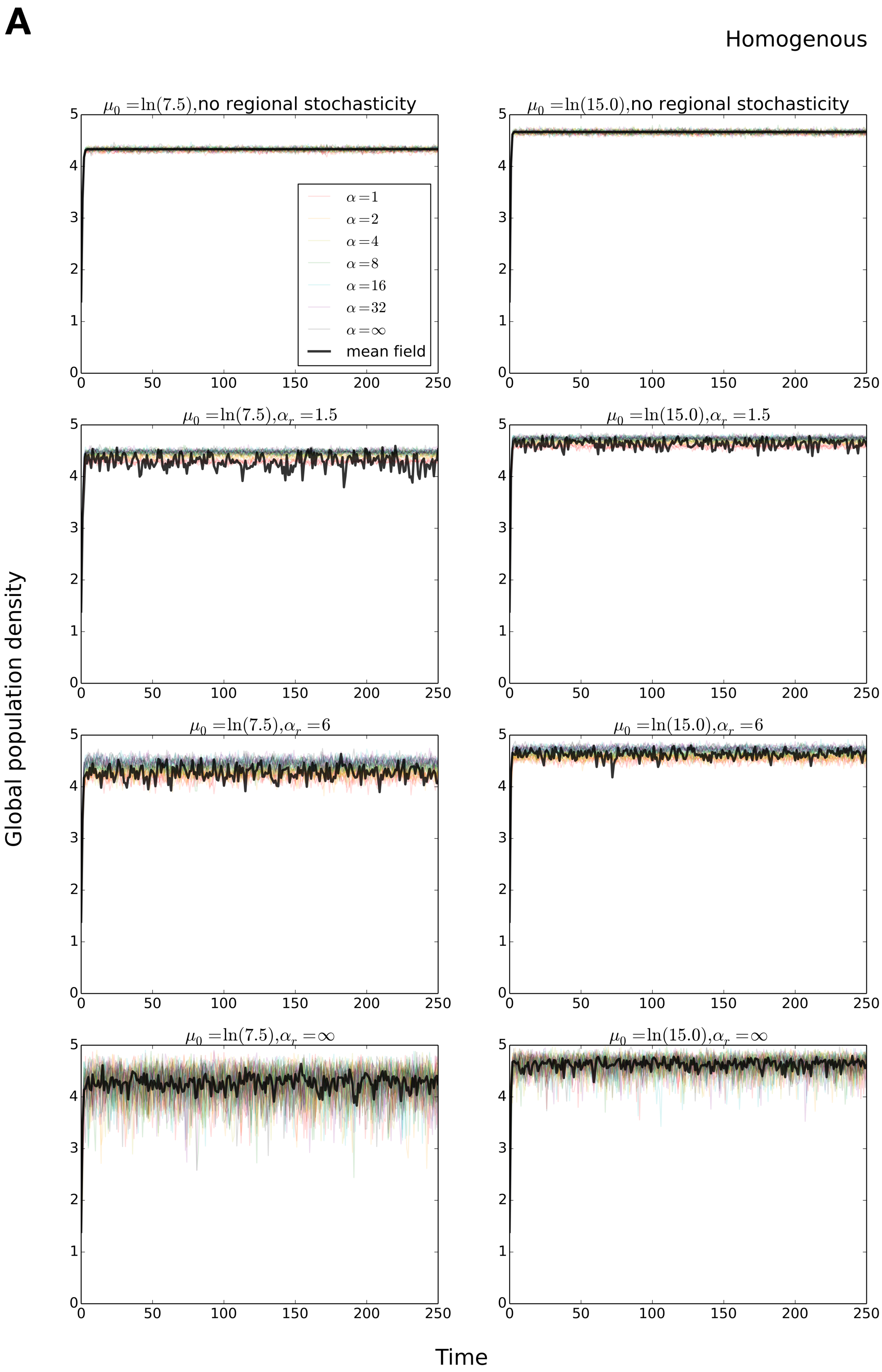

A
Homogenous
$\mu_0 = \ln(7.5)$,no regional stochasticity
$\mu_0 = \ln(15.0)$,no regional stochasticity
$\alpha = 1$
$\alpha = 2$
$\alpha = 4$
$\alpha = 8$
$\alpha = 16$
$\alpha = 32$
$\alpha = \infty$
mean field
$\mu_0 = \ln(7.5), \alpha_r = 1.5$
$\mu_0 = \ln(15.0), \alpha_r = 1.5$
$\mu_0 = \ln(7.5), \alpha_r = 6$
$\mu_0 = \ln(15.0), \alpha_r = 6$
$\mu_0 = \ln(7.5), \alpha_r = \infty$
$\mu_0 = \ln(15.0), \alpha_r = \infty$
Global population density
Time

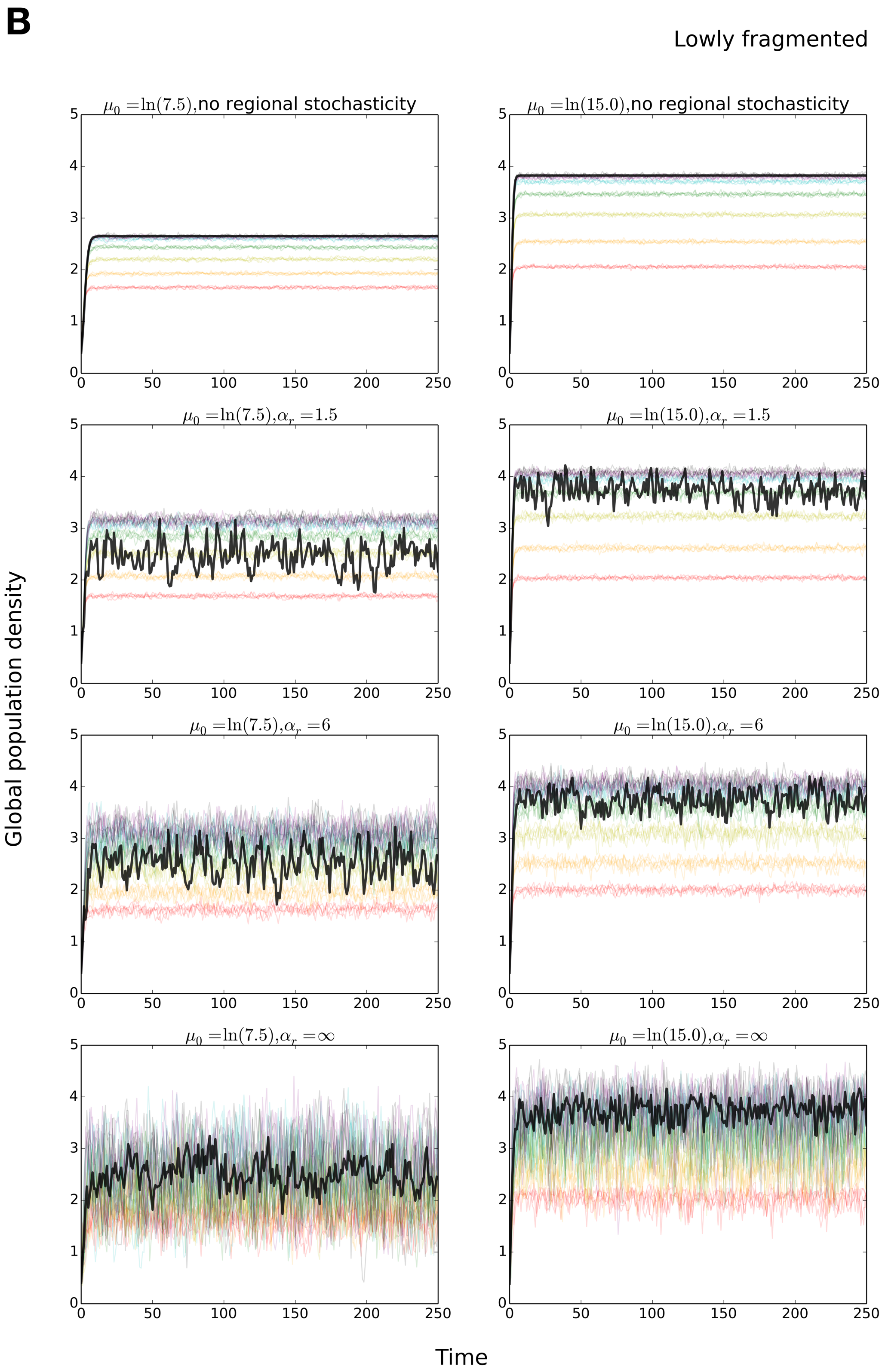
B
Lowly fragmented
$\mu_0 = \ln(7.5)$,no regional stochasticity
$\mu_0 = \ln(15.0)$,no regional stochasticity
$\mu_0 = \ln(7.5), \alpha_r = 1.5$
$\mu_0 = \ln(15.0), \alpha_r = 1.5$
$\mu_0 = \ln(7.5), \alpha_r = 6$
$\mu_0 = \ln(15.0), \alpha_r = 6$
$\mu_0 = \ln(7.5), \alpha_r = \infty$
$\mu_0 = \ln(15.0), \alpha_r = \infty$
Global population density
Time
0
1
2
3
4
5
0
50
100
150
200
250

C

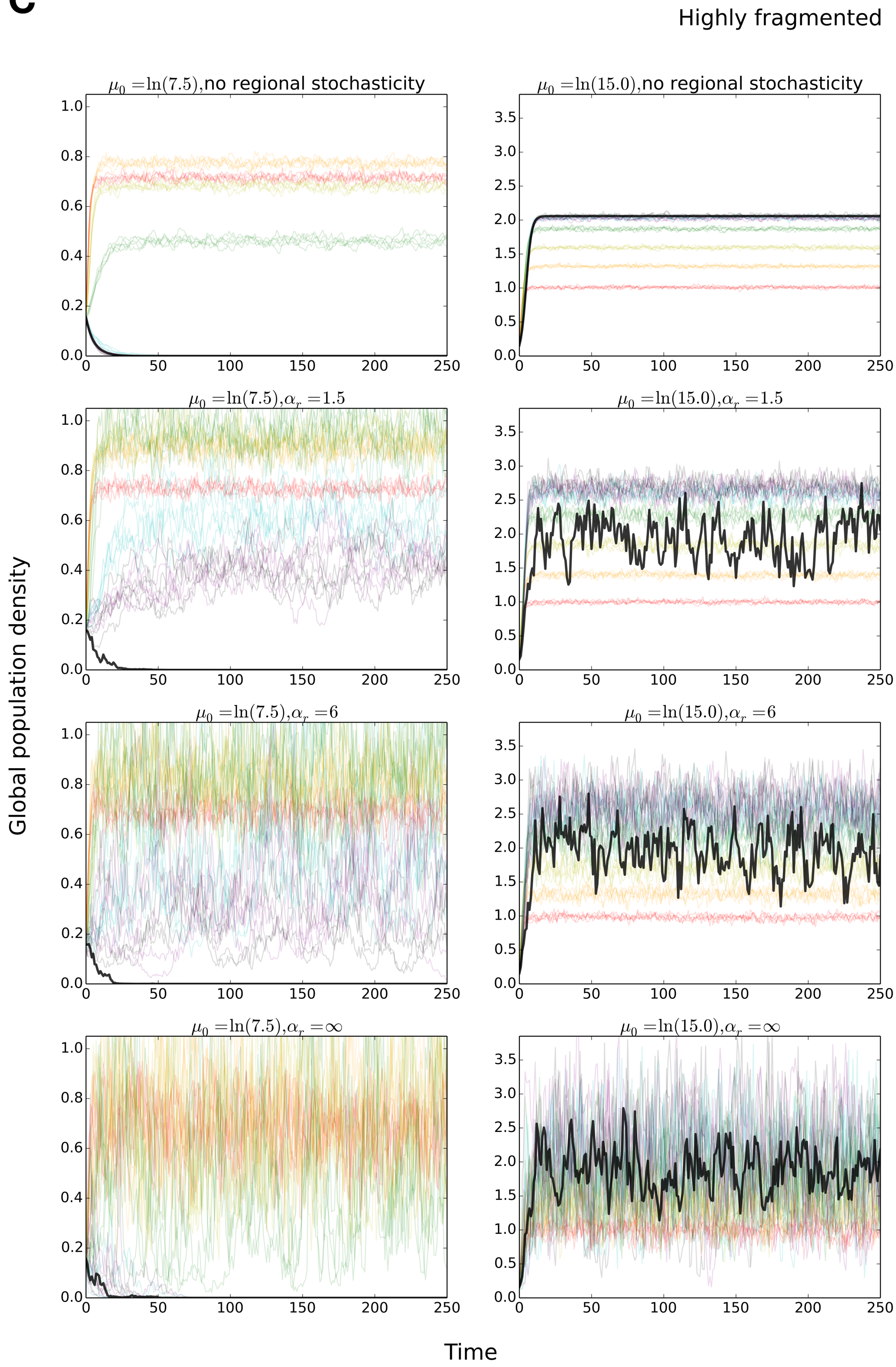

Highly fragmented
$\mu_0 = \ln(7.5)$,no regional stochasticity
$\mu_0 = \ln(15.0)$,no regional stochasticity
$\mu_0 = \ln(7.5), \alpha_r = 1.5$
$\mu_0 = \ln(15.0), \alpha_r = 1.5$
$\mu_0 = \ln(7.5), \alpha_r = 6$
$\mu_0 = \ln(15.0), \alpha_r = 6$
$\mu_0 = \ln(7.5), \alpha_r = \infty$
$\mu_0 = \ln(15.0), \alpha_r = \infty$
Global population density
Time

D

Regular grid

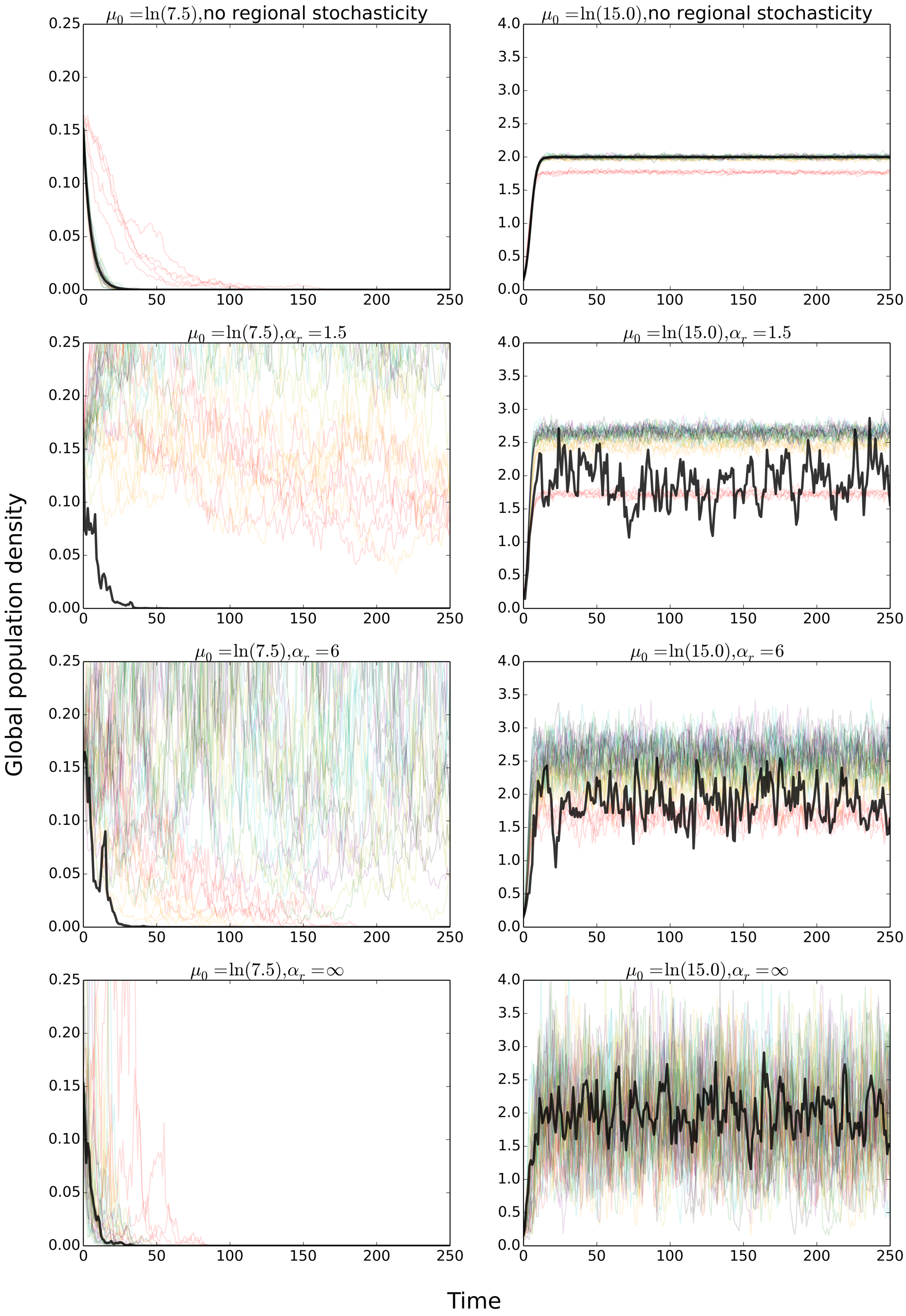

Fig S3. Time series of population densities on four landscapes: a) homogenous, b) lowly fragmented, as pictured in Fig 1b.i, c) highly fragmented, as pictured in Fig 1c.i, and d) regular grid. The model is evaluated in the presence and absence of regional stochasticity under two mean fecundity rates μ_0. Spatial scales of dispersal and competition α and regional stochasticity α_r were varied independently from 1 to ∞. Thick black lines show time-invariant (top rows) and time-variant mean-field predictions. Five simulation iterations were run per parameter set, each initialized with 5000 spatially randomized individuals and tracked for 250 generations. Variance of regional stochasticity $\sigma_r^2 = 0.5$; competition strength $b = 0.2$.

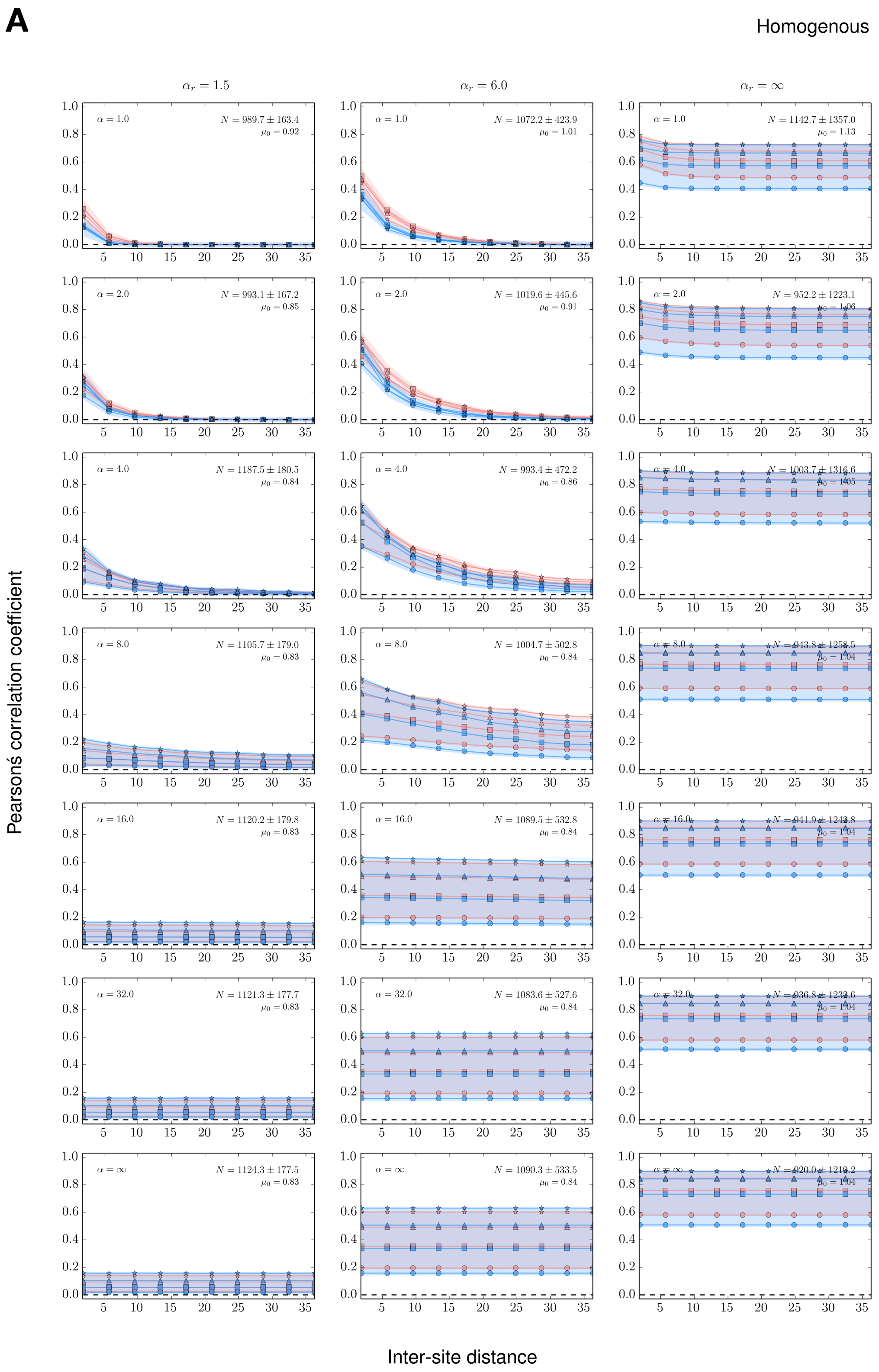
A
Homogenous
$\alpha_r = 1.5$
$\alpha_r = 6.0$
$\alpha_r = \infty$
Pearsonś correlation coefficient
Inter-site distance
$\alpha = 1.0$ $N = 989.7 \pm 163.4$ $\mu_0 = 0.92$
$\alpha = 1.0$ $N = 1072.2 \pm 423.9$ $\mu_0 = 1.01$
$\alpha = 1.0$ $N = 1142.7 \pm 1357.0$ $\mu_0 = 1.13$
$\alpha = 2.0$ $N = 993.1 \pm 167.2$ $\mu_0 = 0.85$
$\alpha = 2.0$ $N = 1019.6 \pm 445.6$ $\mu_0 = 0.91$
$\alpha = 2.0$ $N = 952.2 \pm 1223.1$ $\mu_0 = 1.06$
$\alpha = 4.0$ $N = 1187.5 \pm 180.5$ $\mu_0 = 0.84$
$\alpha = 4.0$ $N = 993.4 \pm 472.2$ $\mu_0 = 0.86$
$\alpha = 4.0$ $N = 1003.7 \pm 1316.6$ $\mu_0 = 1.05$
$\alpha = 8.0$ $N = 1105.7 \pm 179.0$ $\mu_0 = 0.83$
$\alpha = 8.0$ $N = 1004.7 \pm 502.8$ $\mu_0 = 0.84$
$\alpha = 8.0$ $N = 943.8 \pm 1258.5$ $\mu_0 = 1.04$
$\alpha = 16.0$ $N = 1120.2 \pm 179.8$ $\mu_0 = 0.83$
$\alpha = 16.0$ $N = 1089.5 \pm 532.8$ $\mu_0 = 0.84$
$\alpha = 16.0$ $N = 941.9 \pm 1242.8$ $\mu_0 = 1.04$
$\alpha = 32.0$ $N = 1121.3 \pm 177.7$ $\mu_0 = 0.83$
$\alpha = 32.0$ $N = 1083.6 \pm 527.6$ $\mu_0 = 0.84$
$\alpha = 32.0$ $N = 936.8 \pm 1232.6$ $\mu_0 = 1.04$
$\alpha = \infty$ $N = 1124.3 \pm 177.5$ $\mu_0 = 0.83$
$\alpha = \infty$ $N = 1090.3 \pm 533.5$ $\mu_0 = 0.84$
$\alpha = \infty$ $N = 920.0 \pm 1219.2$ $\mu_0 = 1.04$

B

Lowly fragmented

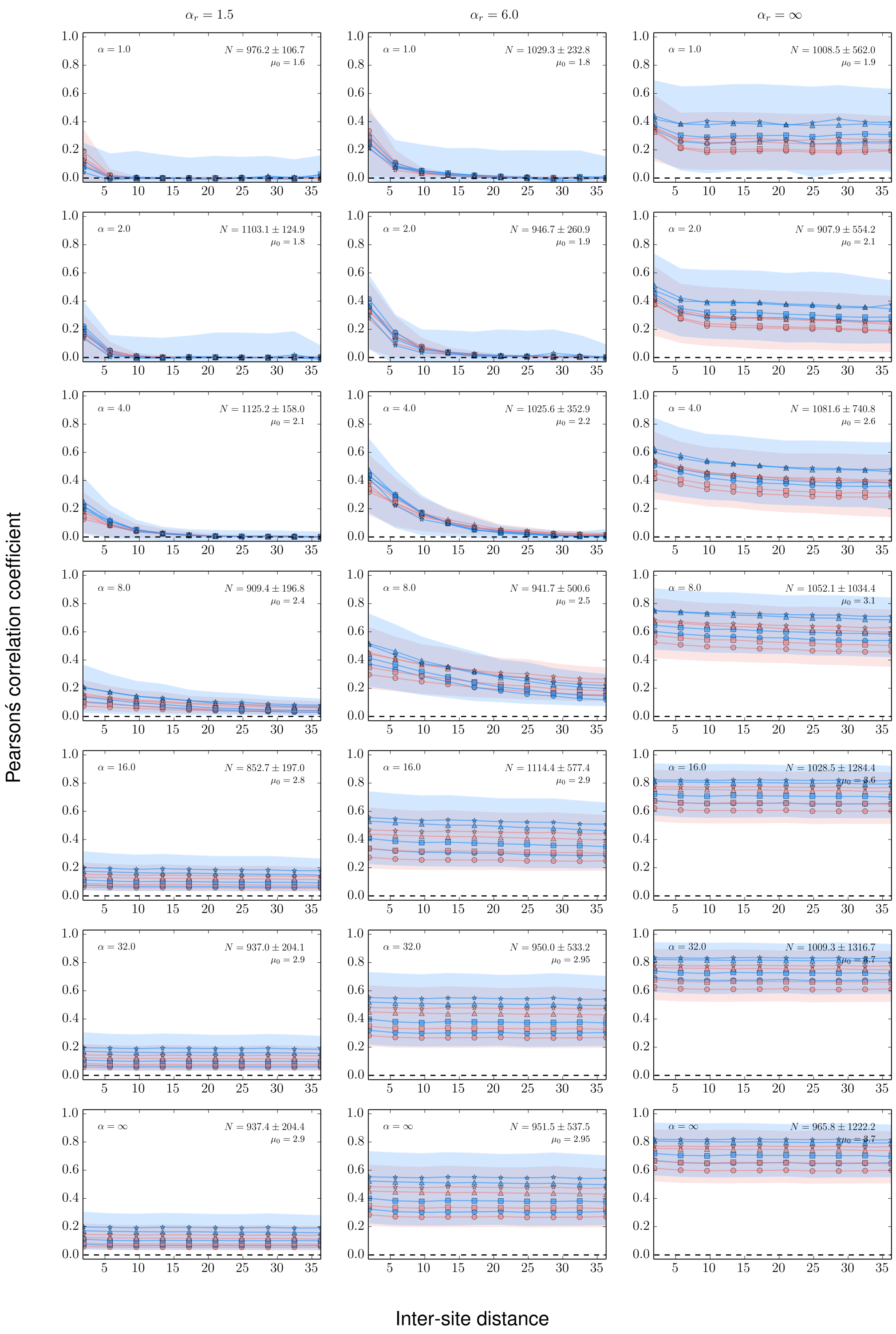

C

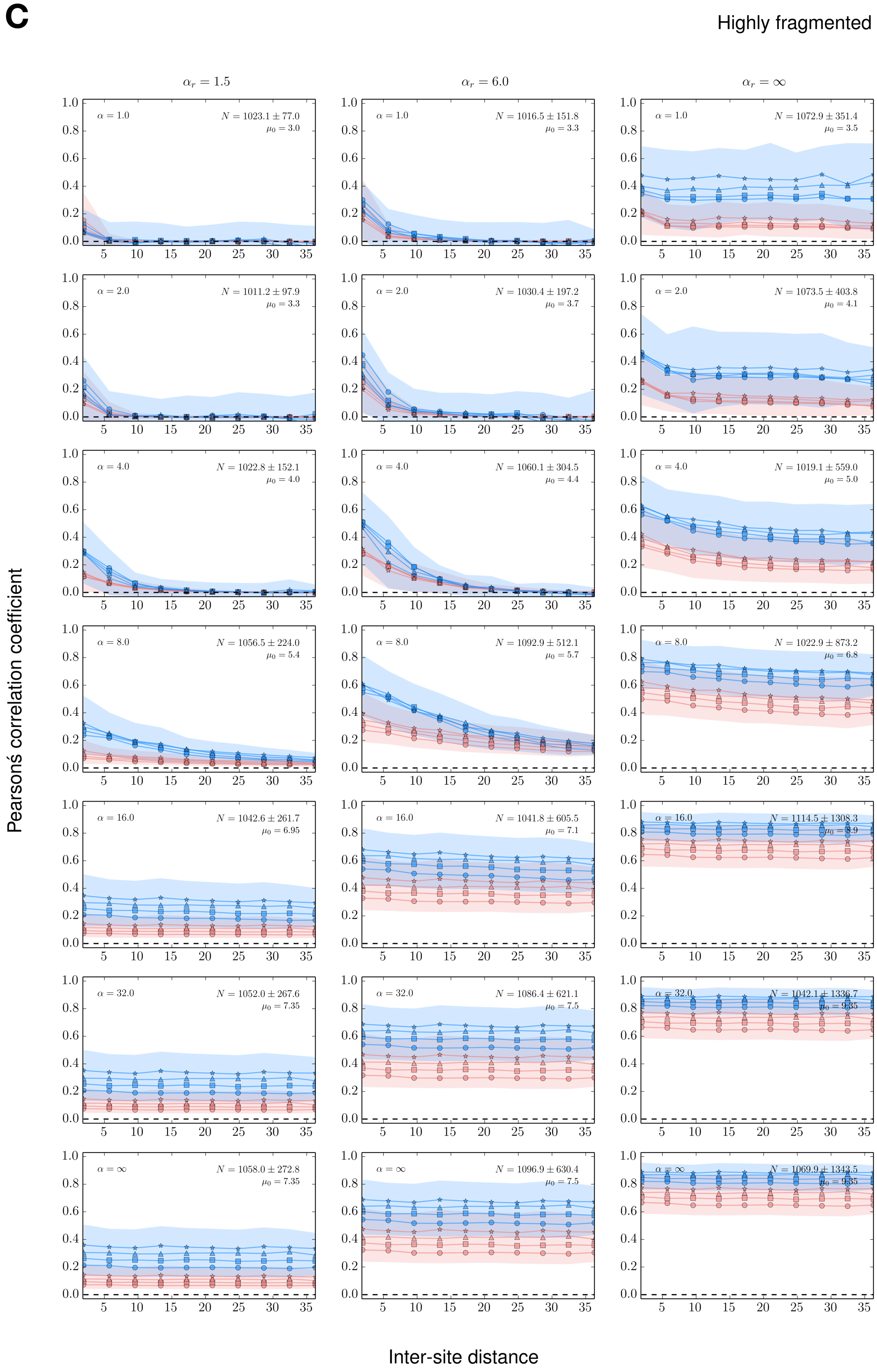
Highly fragmented
$\alpha_r = 1.5$
$\alpha_r = 6.0$
$\alpha_r = \infty$
$\alpha = 1.0$ $N = 1023.1 \pm 77.0$ $\mu_0 = 3.0$
$\alpha = 1.0$ $N = 1016.5 \pm 151.8$ $\mu_0 = 3.3$
$\alpha = 1.0$ $N = 1072.9 \pm 351.4$ $\mu_0 = 3.5$
$\alpha = 2.0$ $N = 1011.2 \pm 97.9$ $\mu_0 = 3.3$
$\alpha = 2.0$ $N = 1030.4 \pm 197.2$ $\mu_0 = 3.7$
$\alpha = 2.0$ $N = 1073.5 \pm 403.8$ $\mu_0 = 4.1$
$\alpha = 4.0$ $N = 1022.8 \pm 152.1$ $\mu_0 = 4.0$
$\alpha = 4.0$ $N = 1060.1 \pm 304.5$ $\mu_0 = 4.4$
$\alpha = 4.0$ $N = 1019.1 \pm 559.0$ $\mu_0 = 5.0$
$\alpha = 8.0$ $N = 1056.5 \pm 224.0$ $\mu_0 = 5.4$
$\alpha = 8.0$ $N = 1092.9 \pm 512.1$ $\mu_0 = 5.7$
$\alpha = 8.0$ $N = 1022.9 \pm 873.2$ $\mu_0 = 6.8$
$\alpha = 16.0$ $N = 1042.6 \pm 261.7$ $\mu_0 = 6.95$
$\alpha = 16.0$ $N = 1041.8 \pm 605.5$ $\mu_0 = 7.1$
$\alpha = 16.0$ $N = 1114.5 \pm 1308.3$ $\mu_0 = 8.9$
$\alpha = 32.0$ $N = 1052.0 \pm 267.6$ $\mu_0 = 7.35$
$\alpha = 32.0$ $N = 1086.4 \pm 621.1$ $\mu_0 = 7.5$
$\alpha = 32.0$ $N = 1042.1 \pm 1336.7$ $\mu_0 = 9.35$
$\alpha = \infty$ $N = 1058.0 \pm 272.8$ $\mu_0 = 7.35$
$\alpha = \infty$ $N = 1096.9 \pm 630.4$ $\mu_0 = 7.5$
$\alpha = \infty$ $N = 1069.9 \pm 1343.5$ $\mu_0 = 9.35$
Pearsonś correlation coefficient
Inter-site distance

D

Regular grid

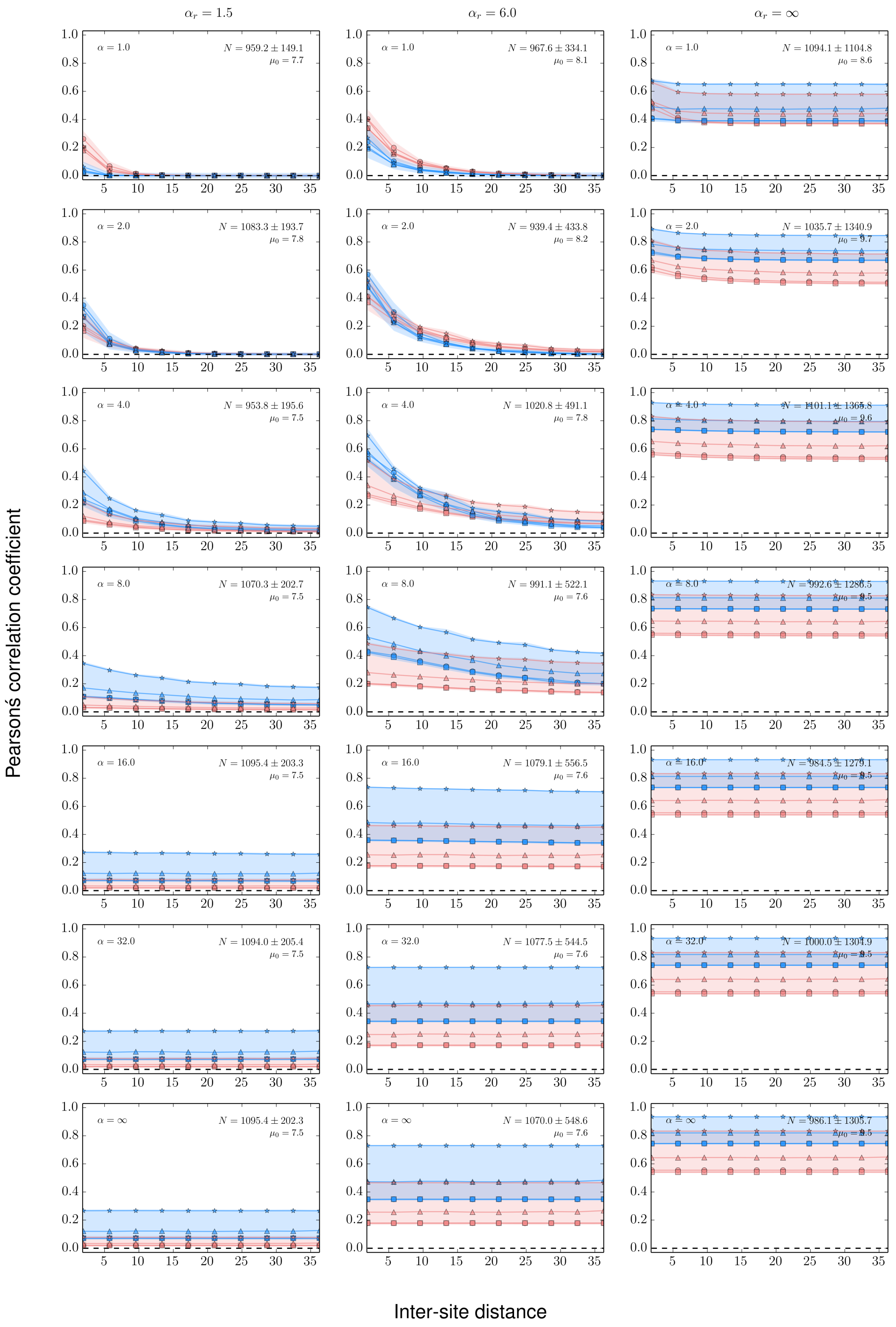

Fig S4. Spatial synchrony on four landscapes: a) homogenous, b) lowly fragmented, as pictured in Fig 1b.i, c) highly fragmented, as pictured in Fig 1c.i, and d) regular grid. Synchrony was measured by the pairwise correlations in local patch occupancy (red) and local abundance (blue) at the final generation. The markers denote the correlation coefficients between sampling sites of lengths m (2: circle; 3: square; 4: triangle; 5: star) averaged over distance bins; the colorized boundaries show the maximal bounds of standard deviations. Mean fecundity rates μ_0 were adjusted to maintain a near-constant mean global population size N (1000$\pm$200) across all combinations of landscape structure, spatial scale of dispersal and competition α, and spatial scale of regional stochasticity α_r. 10000 simulation iterations were run per parameter set, each initialized with 5000 randomized individuals and tracked for 50 generations. The broken horizontal lines mark the correlational limit where population dynamics are asynchronous. Variance of regional stochasticity $\sigma_r^2 = 0.5$; competition strength $b = 0.2$.

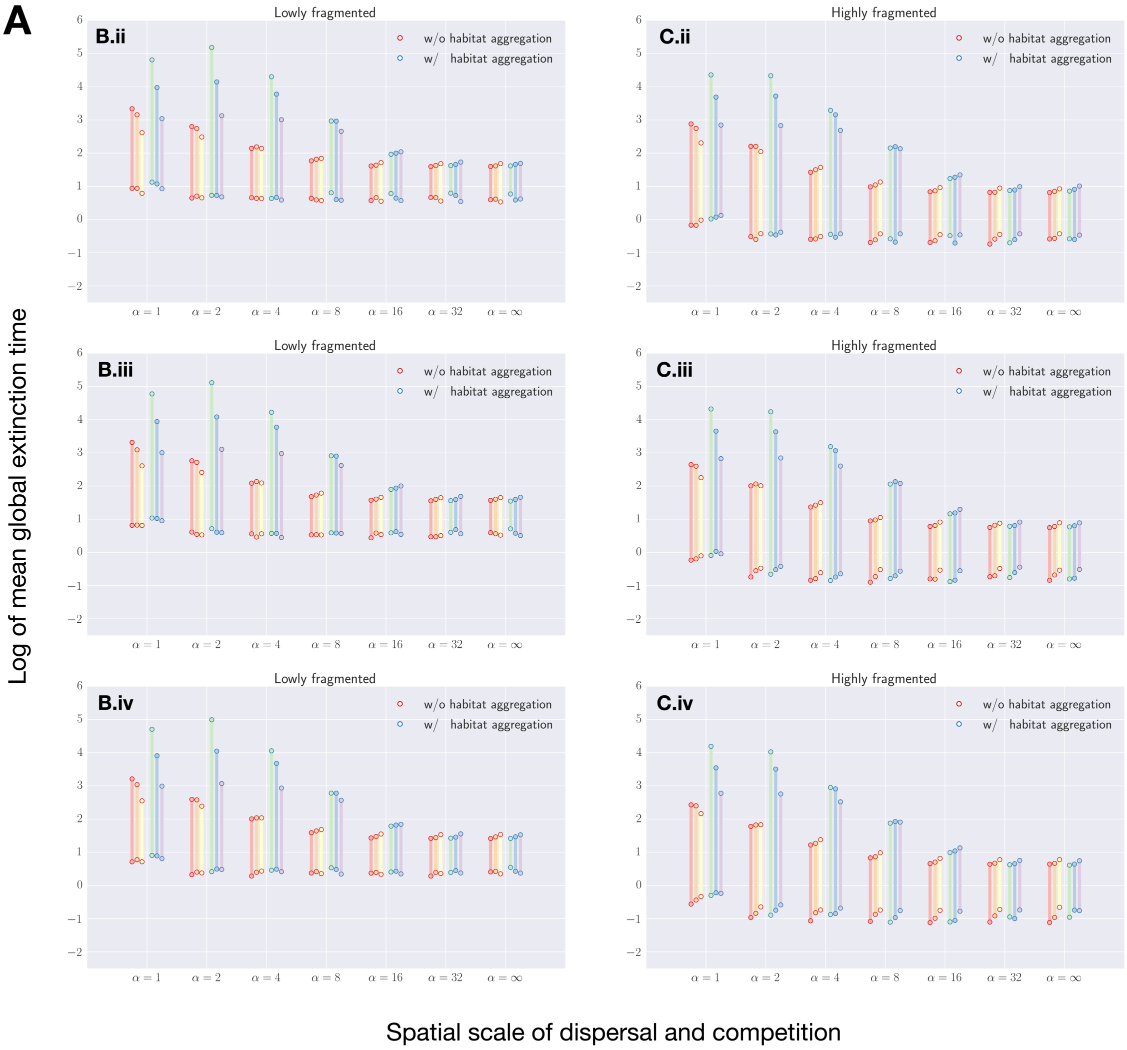
A
B.ii
Lowly fragmented
C.ii
Highly fragmented
B.iii
Lowly fragmented
C.iii
Highly fragmented
B.iv
Lowly fragmented
C.iv
Highly fragmented
w/o habitat aggregation
w/ habitat aggregation
$\alpha = 1$
$\alpha = 2$
$\alpha = 4$
$\alpha = 8$
$\alpha = 16$
$\alpha = 32$
$\alpha = \infty$
Log of mean global extinction time
Spatial scale of dispersal and competition

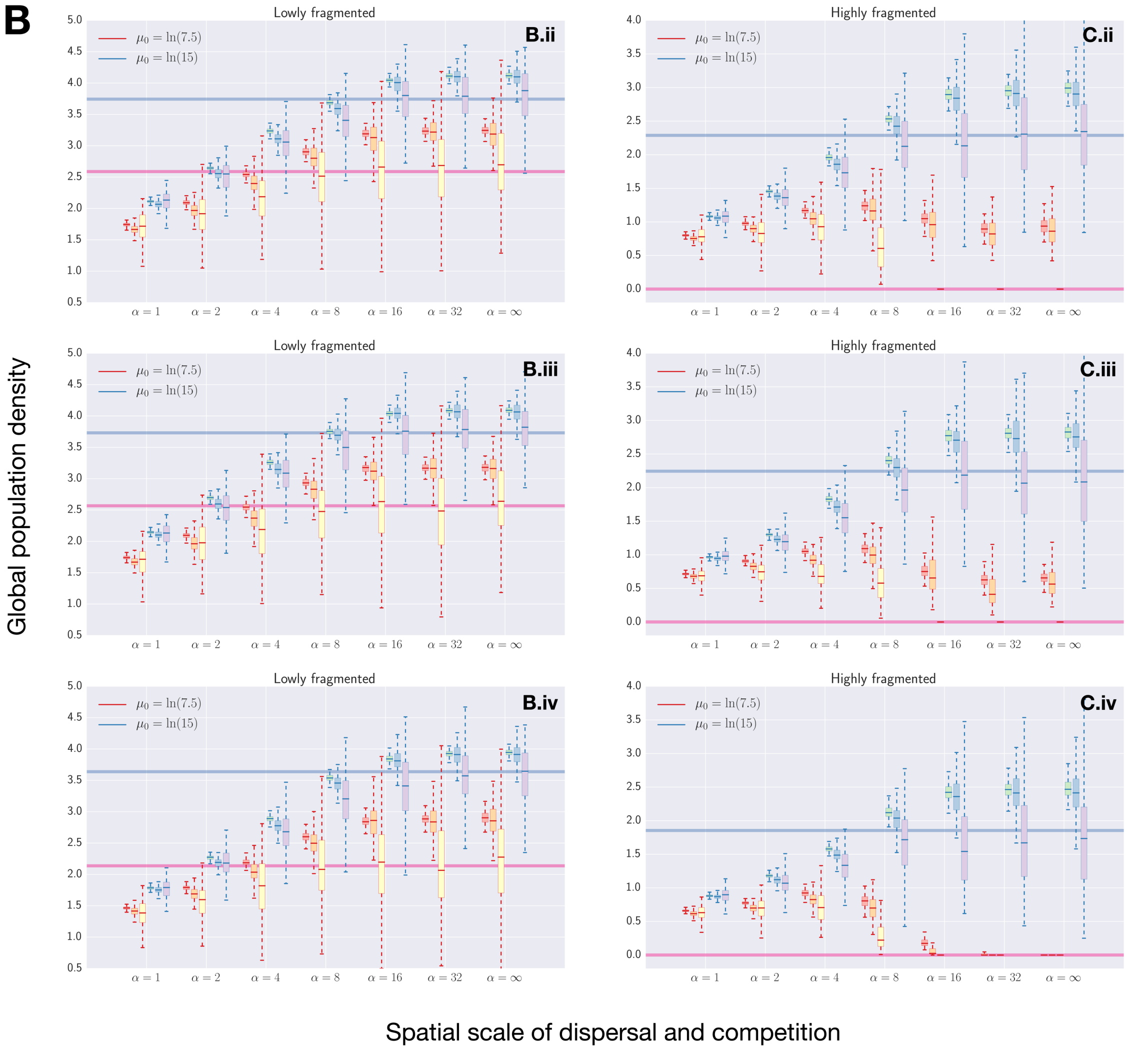
B
Global population density
Lowly fragmented
Highly fragmented
B.ii
B.iii
B.iv
C.ii
C.iii
C.iv
$\mu_0 = \ln(7.5)$
$\mu_0 = \ln(15)$
$\alpha = 1$
$\alpha = 2$
$\alpha = 4$
$\alpha = 8$
$\alpha = 16$
$\alpha = 32$
$\alpha = \infty$
Spatial scale of dispersal and competition

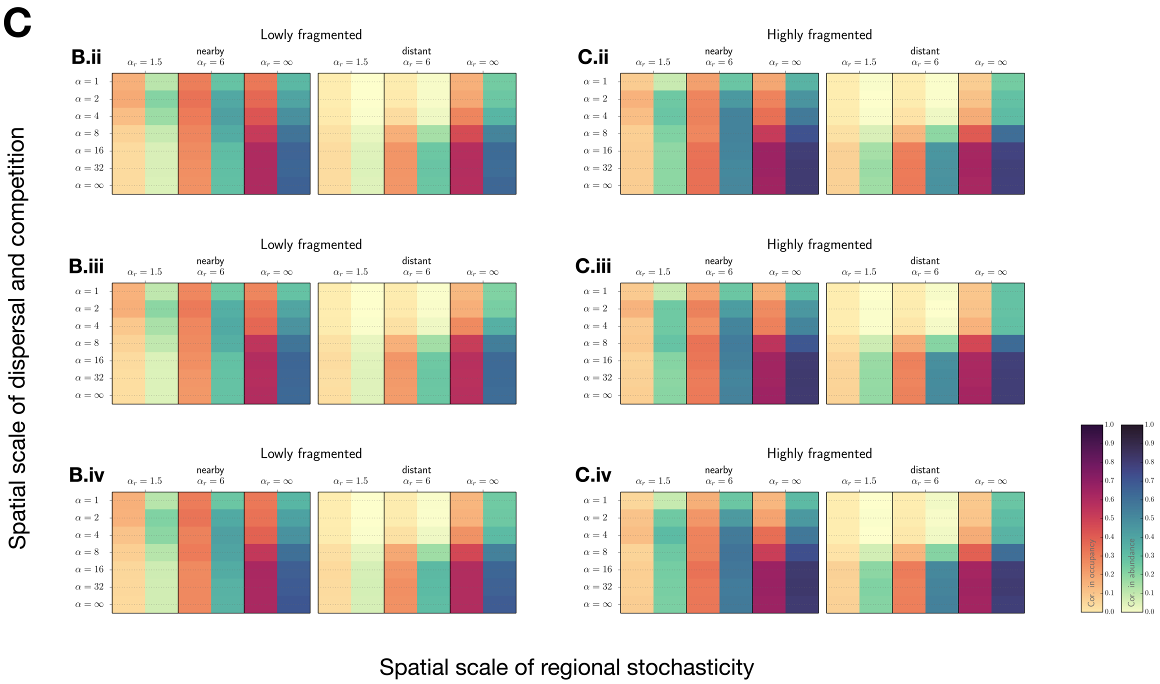

Fig S5. Metapopulation dynamics on lowly and highly fragmented landscapes, as pictured in Fig 1b.ii-iv and c.ii-iv, evaluated based on a) mean time to global extinction, b) global population density, and c) spatial synchrony. The parameter settings are the same as those in Figs 2-4. Results are qualitatively consistent across randomized fragmentation scenarios.

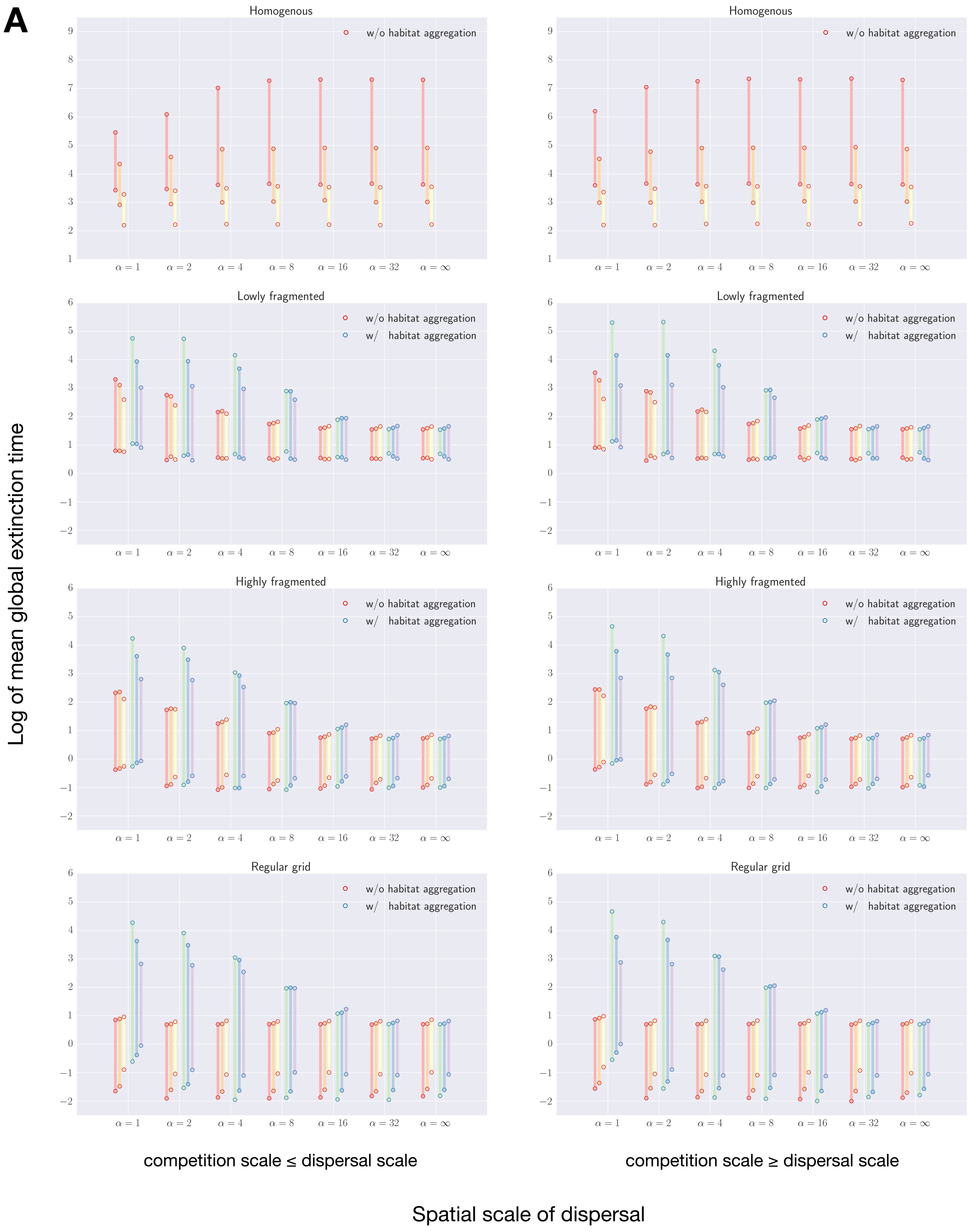

A
Log of mean global extinction time
Homogenous
w/o habitat aggregation
Lowly fragmented
w/ habitat aggregation
Highly fragmented
Regular grid
$\alpha = 1$
$\alpha = 2$
$\alpha = 4$
$\alpha = 8$
$\alpha = 16$
$\alpha = 32$
$\alpha = \infty$
competition scale ≤ dispersal scale
competition scale ≥ dispersal scale
Spatial scale of dispersal

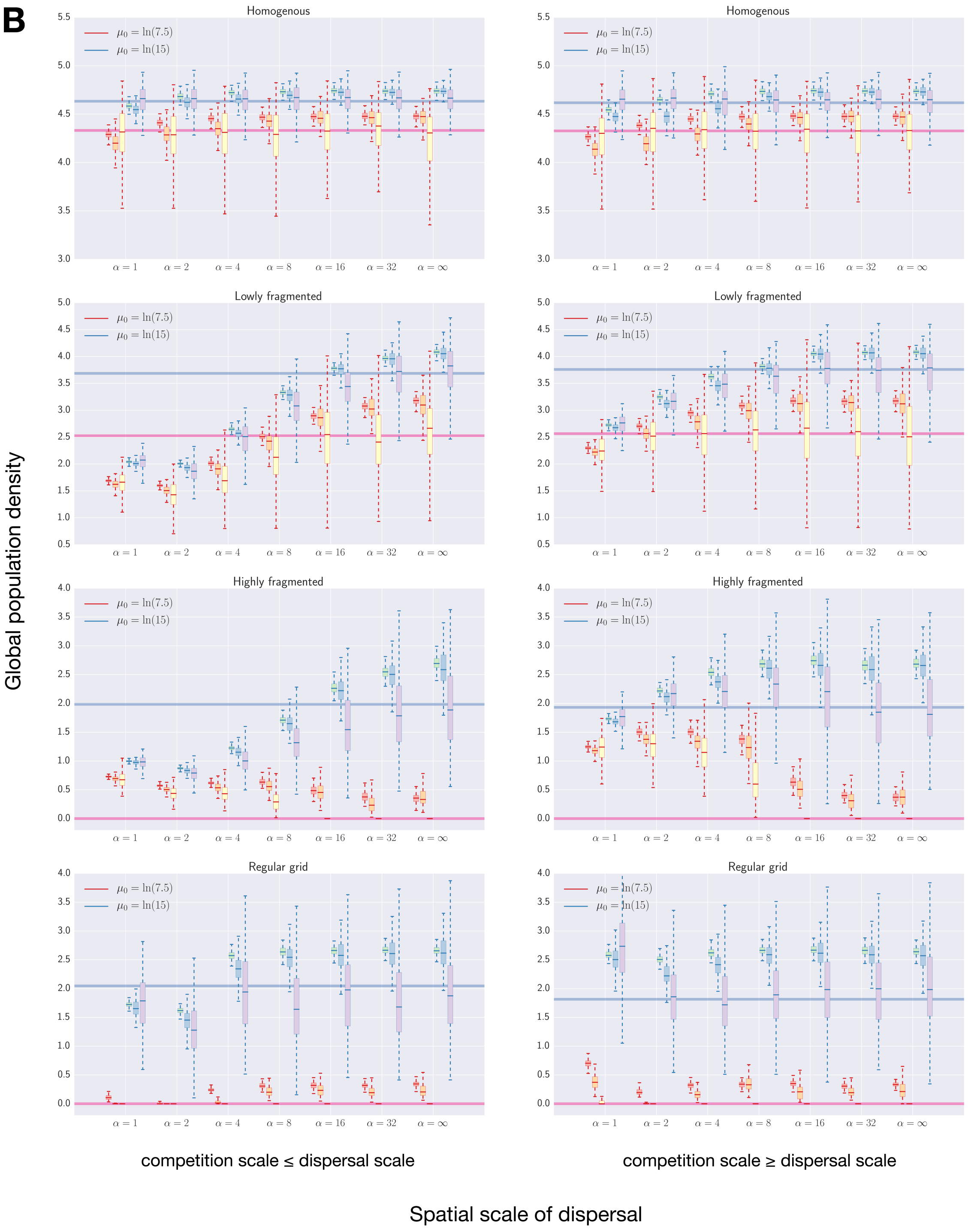
B
Homogenous
Lowly fragmented
Highly fragmented
Regular grid
$\mu_0 = \ln(7.5)$
$\mu_0 = \ln(15)$
$\alpha = 1$
$\alpha = 2$
$\alpha = 4$
$\alpha = 8$
$\alpha = 16$
$\alpha = 32$
$\alpha = \infty$
Global population density
competition scale ≤ dispersal scale
competition scale ≥ dispersal scale
Spatial scale of dispersal

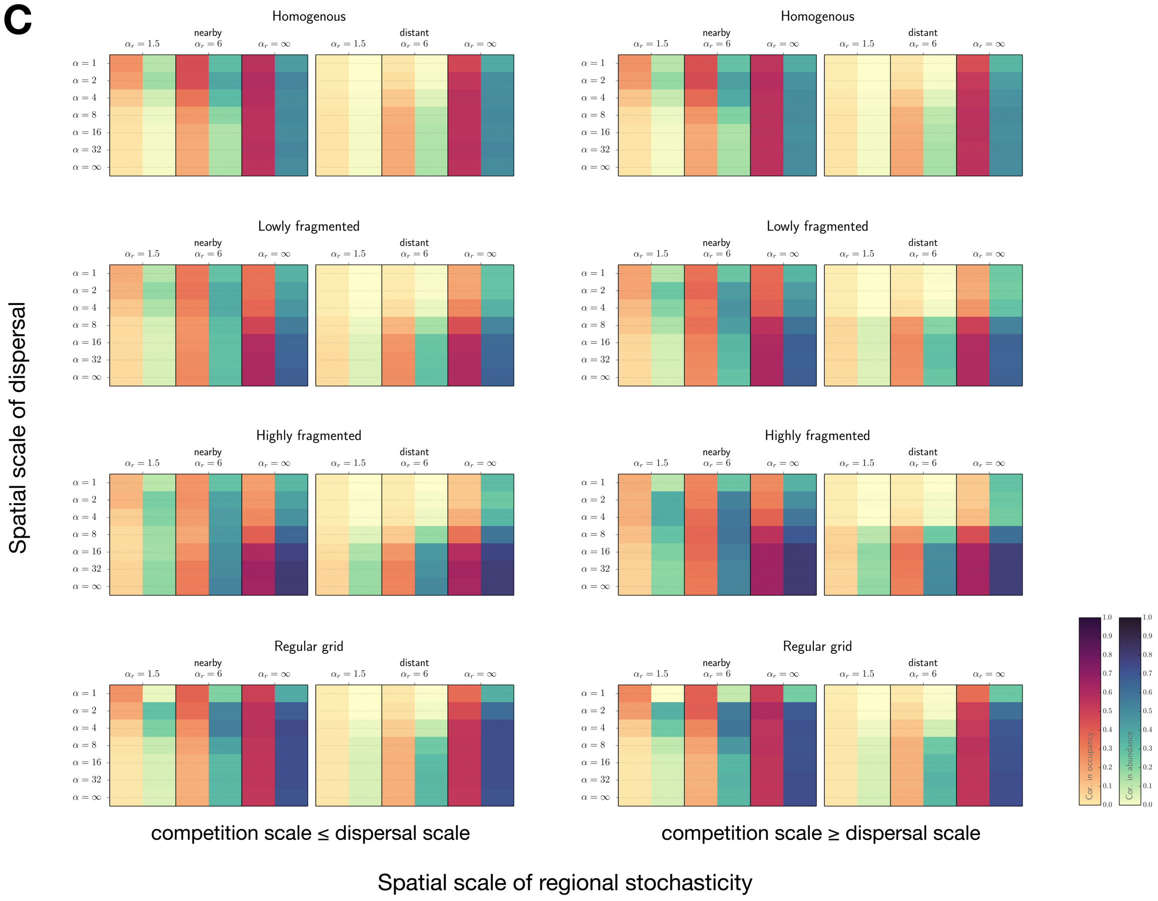

Fig S6. Metapopulation dynamics under various levels of differences between the spatial scales of dispersal and competition, evaluated based on a) mean time to global extinction, b) global population density, and c) spatial synchrony. For each finite dispersal scale α, the competition scale is defined as $\alpha_c = \max(1, \alpha/2)$ (left column) and $\alpha_c = \max(1, 2\alpha)$ (right column). The landscapes and the remaining parameter settings are the same as those in Figs 2-4. Patterns in the main results are reproduced despite scale discrepancies.
